# Supplementary material for: Therapeutic targeting ERRγ suppresses metastasis via extracellular matrix remodeling in small cell lung cancer
Source: EMBO Mol Med. 2024 Jul 31;16(9):2043–59. doi: 10.1038/s44321-024-00108-z (PMC11393344; doi:10.1038/s44321-024-00108-z)
Supplement: Supplementary file 1 — Appendix [file 44321_2024_108_MOESM1_ESM.pdf]

Appendix

Therapeutic targeting ERRγ suppresses metastasis via extracellular matrix remodeling in small cell lung cancer

Hong Wang[1,9], Huizi Sun[1,9], Jie Huang[2,9], Zhenhua Zhang[1], Guodi Cai[1], Chaofan Wang[3], Kai Xiao[4], Xiaofeng Xiong[1], Jian Zhang[5], Peiqing Liu[1, 6, 7], Xiaoyun Lu[3*], Weineng Feng[8*], Junjian Wang[1, 6, 7*]

***Corresponding author**

wangjj87@mail.sysu.edu.cn (Junjian Wang) or fwneng@fsyyy.com (Weineng Feng) or luxy2016@jnu.edu.cn (Xiaoyun Lu)

This PDF file includes:

Appendix Figure S1. ESRRG is overexpressed in SCLC and associated with SCLC metastasis...2

Appendix Figure S2. ERRγ is a major driver of SCLC cell survival and tumor tumorigenesis...3

Appendix Figure S3. ERRγ promotes SCLC cell invasion and metastasis..............6

Appendix Figure S4. ERRγ inhibition reprograms ECM signaling.......................8

Appendix Figure S5. ERRγ inhibition sensitizes SCLC tumor to chemotherapy.........9

Appendix Table S1. Chemicals...11

Appendix Table S2. Primers for qPCR used in this study...............................12

Appendix Table S3. Antibodies for immunoblotting and immunohistochemistry......13

Appendix Table S4. Primers for ChIP-qPCR used in this study.........................14

Appendix Table S5. Sequences for siRNA used in this study...........................15

Appendix Table S6. Sequences for shRNA used in this study...........................16

Appendix Figure S1

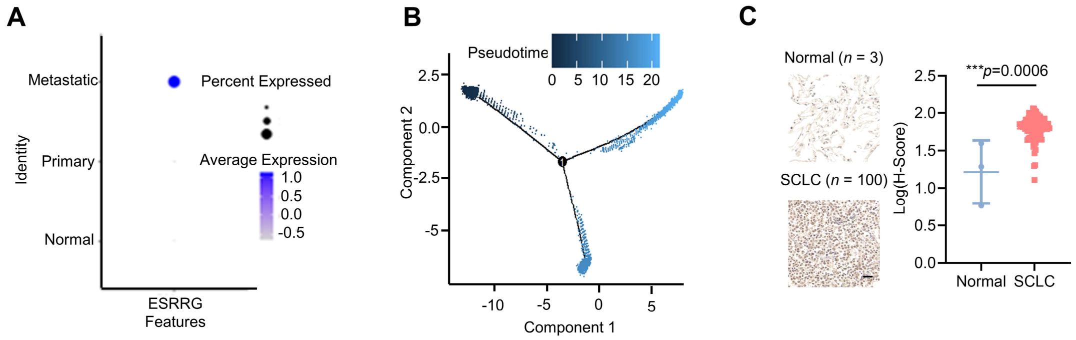

Appendix Figure S1. ESRRG is overexpressed in SCLC and associated with SCLC metastasis.

(A) Dot plot of the mean level of ESRRG (dot intensity, blue scale) and percent of cells in population with detected expression (dot size), corresponding to the normal lung tissues, primary tumors and metastatic tumors (*n* = 21 patients).

(B) Monocle pseudotime trajectory analysis of malignant SCLC cells (*n* = 21 patients).

(C) Representative images and statistical diagram of average Log(H-Score) from ERRγ immunohistochemistry of normal lung tissues (*n* = 3) and SCLC tissues (*n* = 100) specimens. Scale bar, 20 μm.

Data information: Data represent different numbers (*n*) of biological replicates. Data shown in (C) are presented as mean ± s.d. Student's *t*-test is used in (C). $^{***}p < 0.001$. Source data are available online for this figure.

Appendix Figure S2

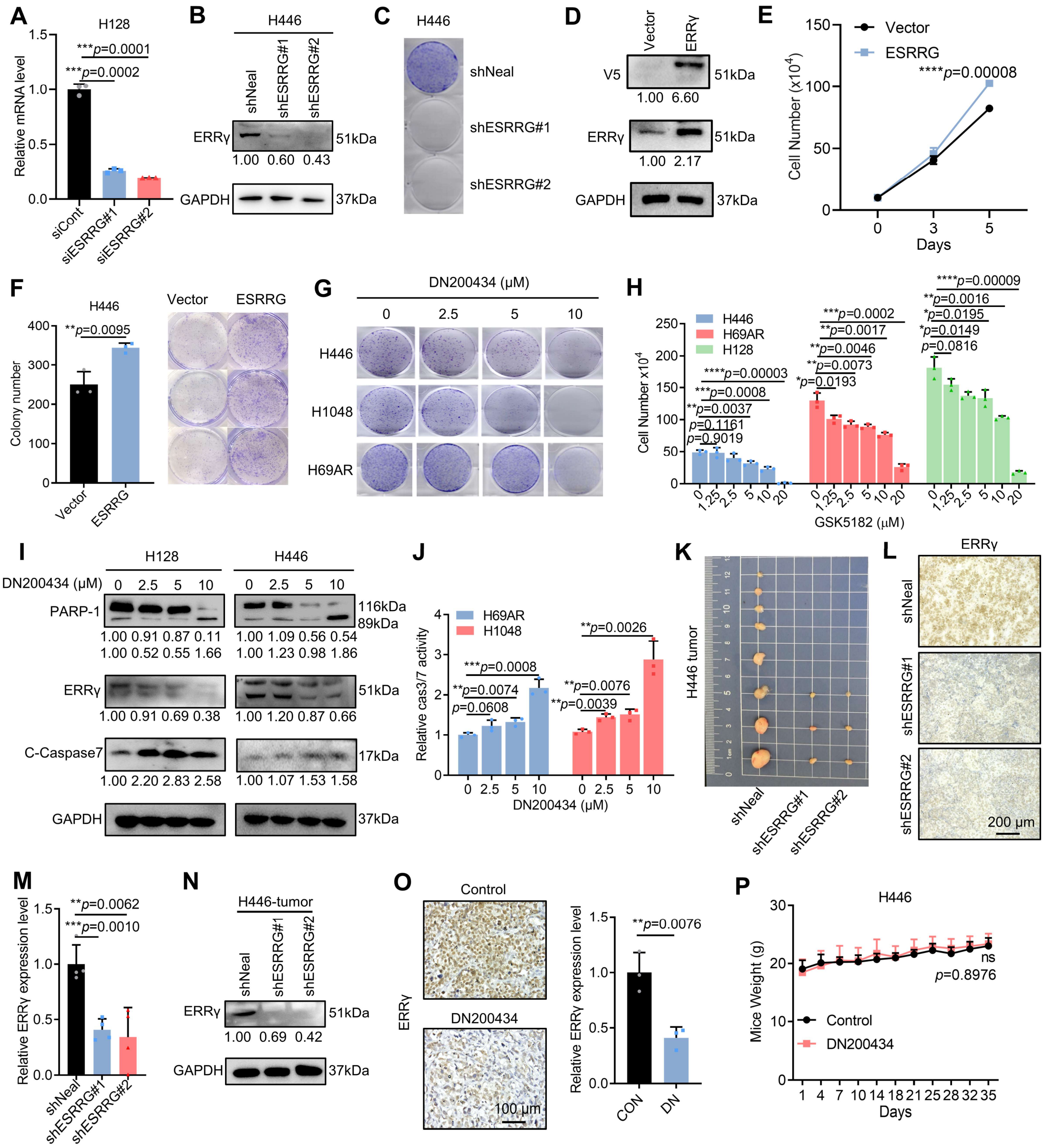

Appendix Figure S2. ERRγ is a major driver of SCLC cell survival and tumor tumorigenesis.

(A) qRT-PCR analysis of ESRRG in H128 cells transfected with siESRRG or vehicle (n = 3 biological replicates).

(B) Immunoblotting analysis of ERRγ protein levels in H446 cells transfected with shRNA against ESRRG.

(C) Representative images of colonies formed by H446 cells transfected with shRNA against ESRRG (n = 3 biological replicates).

(D) Immunoblotting analysis of ERRγ overexpressed H446 cells.

(E) H446 cell was infected by ERRγ overexpressing or control lentiviruses. The number of cells was counted in day 3 and day 5 (n = 3 biological replicates).

(F) Representative images (right) and statistical result (left) of colonies formed by wild-type and ERRγ overexpressed H446 cells (n = 3 biological replicates).

(G) Representative images of colonies formed by SCLC cells treated with DN200434 or vehicle (n = 3 biological replicates).

(H) SCLC cells were treated with vehicle or GSK5182 as indicated. After 96 h, total viable cells were counted (n = 3 biological replicates).

(I) Immunoblotting analysis of apoptosis-related protein levels in SCLC cells treated with DN200434 or vehicle.

(J) The influence of DN200434 on apoptosis were measured by using a luminescent caspase-Glo 3/7 assay kit on H69AR and H1048 cells treated with vehicle or the indicated concentrations of DN200434 for 72 h (n = 3 biological replicates).

(K) Images of H446 tumors transfected with shESRRG (n = 7 mice per group) or vehicle (n = 9 mice per group) for 38 days.

(L) Representative anti-ERRγ immunohistochemistry images of tumor sections from mice transfected with vehicle or shESRRG. Scale bar, 200 µm (n = 3 biological replicates).

(M) Representative IHC statistical result of indicated tumor sections from mice as in Figure (L) (n = 3 biological replicates).

(N) Immunoblotting of ERRγ protein in tumors from H446-bearing mice transfected with vehicle or shESRRG.

(O) Anti-ERRγ immunohistochemistry images (right) and statistical result (left) of tumor sections from mice treated with 50 mg/kg DN200434 or vehicle. Scale bar, 100 µm (n = 3 biological replicates).

(P) Body weight of mice bearing H446 were treated with 50 mg/kg DN200434 or vehicle. Mean mice body weight ± s.e.m. was shown. Significance was calculated using Student's t-test. n = 5 mice per group. ns, not significant.

Data information: Data represent different numbers (n) of biological replicates. Data shown in (A, E-F, H, J, M, O) are presented as mean ± s.d. Data shown in (P) are presented as mean ± s.e.m. Student's t-test is used in (A, E-F, H, J, M, O-P). $^*p < 0.05$, $^{**}p < 0.01$, $^{***}p < 0.001$, $^{****}p < 0.0001$. Source data are available online for this figure.

Appendix Figure S3

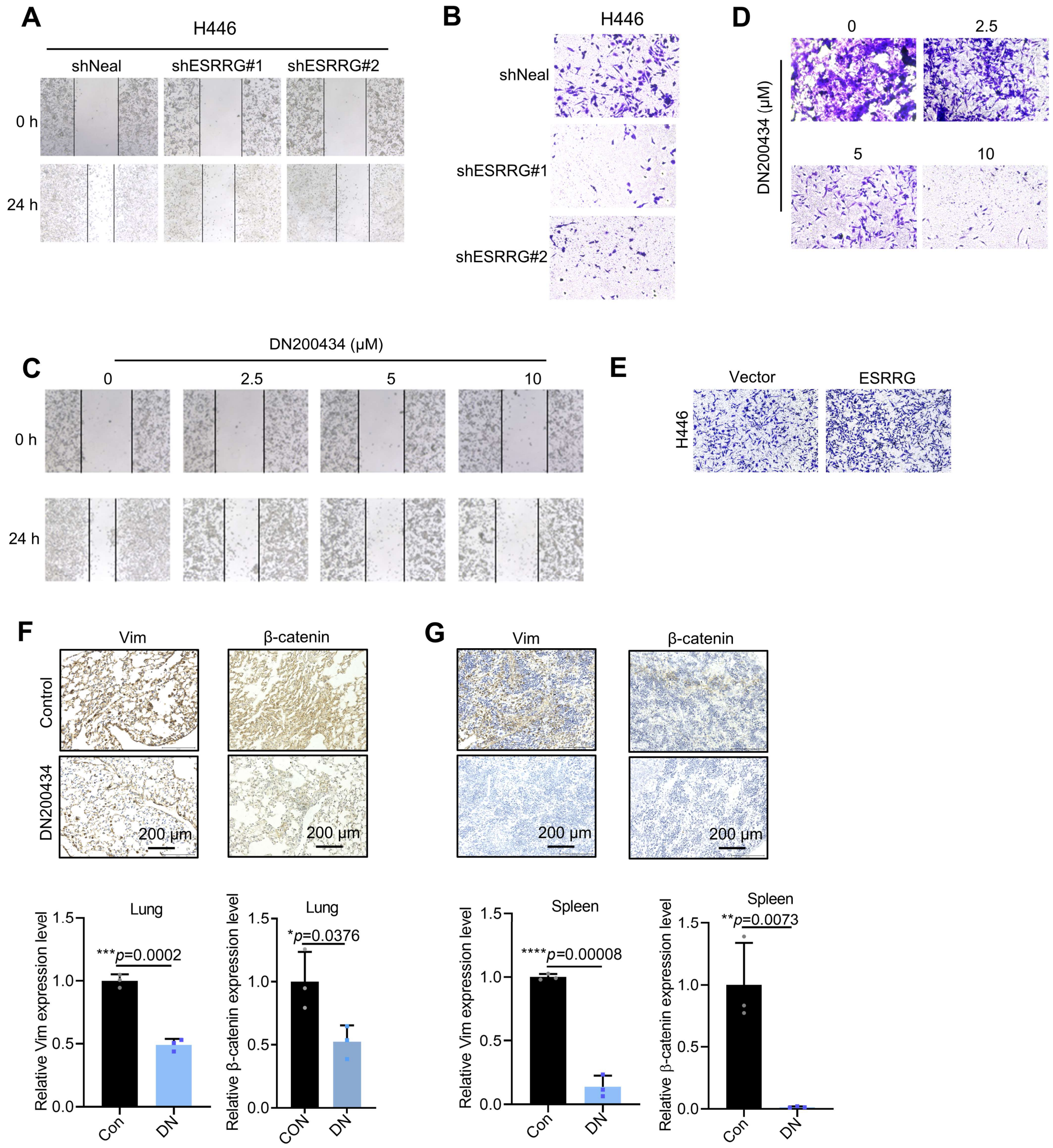

Appendix Figure S3. ERRγ promotes SCLC cell invasion and metastasis.

(A) Representative images of the wound healing assay in H446 cells with or without ESRRG knockdown (n = 3 biological replicates).

(B) Representative images of transwell assays from the indicated groups with or without ESRRG knockdown in H446 cells (n = 3 biological replicates).

(C) Representative images of the wound healing assay in H446 cells treated with vehicle or DN200434 (n = 3 biological replicates).

(D) Representative images of transwell assays from the indicated groups treated with

vehicle or DN200434 in H446 cells (n = 3 biological replicates).

(E) Representative images of transwell assays from wild-type and ERRγ overexpressed H446 cells (n = 3 biological replicates).

(F) Representative images (top) and statistical diagram (bottom) of IHC staining for Vimentin and β-catenin of lung tissues from Figure (3H). Scale bar, 200 μm (n = 3 biological replicates).

(G) Representative images (top) and statistical diagram (bottom) of IHC staining for Vimentin and β-catenin of spleen tissues from Figure (3F). Scale bar, 200 μm (n = 3 biological replicates).

Data information: Data represent different numbers (n) of biological replicates. Data shown in (F-G) are presented as mean ± s.d. Student's t-test is used in (F-G). $^{*}p < 0.05$, $^{**}p < 0.01$, $^{***}p < 0.001$, $^{****}p < 0.0001$. Source data are available online for this figure.

Appendix Figure S4

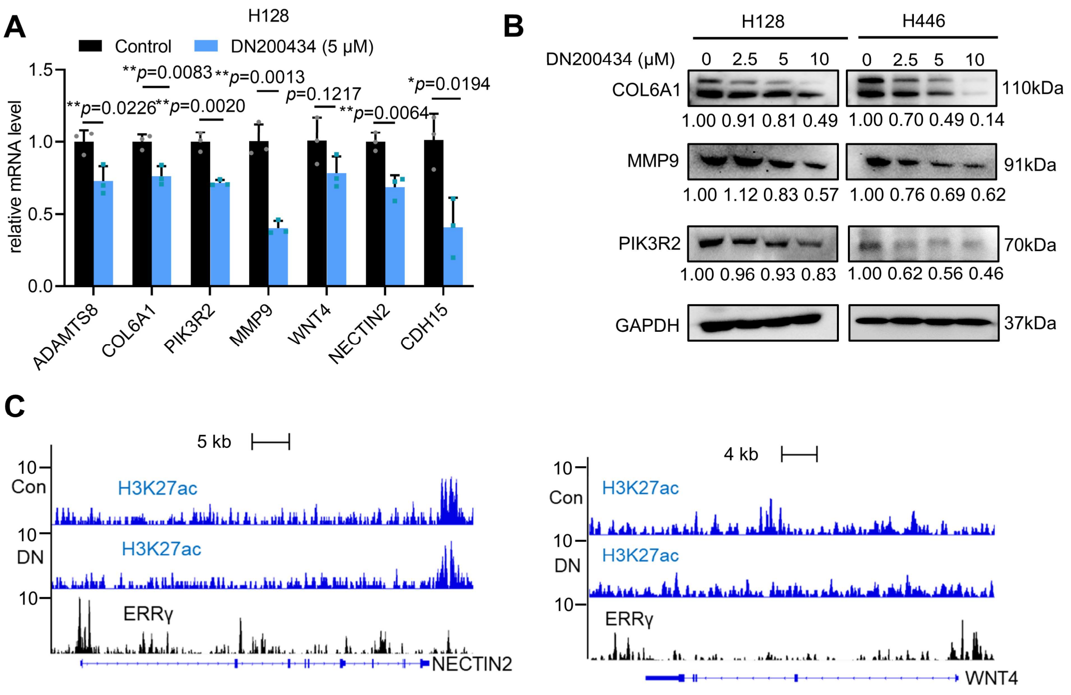

Appendix Figure S4. ERRγ inhibition reprograms ECM signaling.

(A) qRT-PCR analysis of the indicated genes in H128 cells treated with DN200434 or vehicle (n = 3 biological replicates).

(B) Immunoblotting of indicated proteins in H446 cells and H128 cells, both treated with vehicle or DN200434.

(C) ChIP-Seq profiles of H3K27ac and public ChIP-Seq profiles of ERRγ binding around the center of peak regions on genes involved in ECM remodeling pathway.

Data information: Data represent different numbers (n) of biological replicates. Data shown in (A) are presented as mean ± s.d. Student's t-test is used in (A). $*p < 0.05$, $**p < 0.01$. ns, not significant. Source data are available online for this figure.

Appendix Figure S5

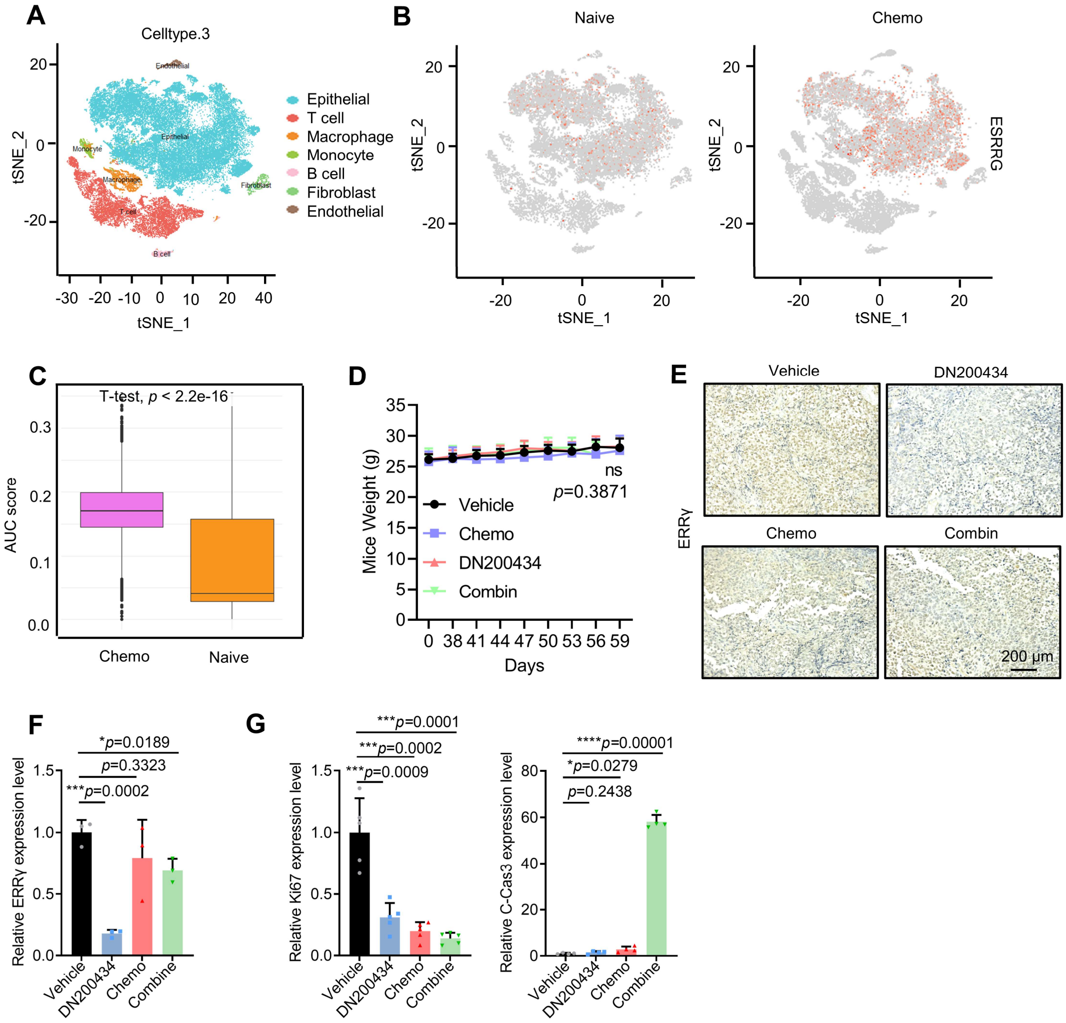

Appendix Figure S5. ERRγ inhibition sensitizes SCLC tumor to chemotherapy.

(A) UMAP visualization of all cells clustered and color coded by cell type.

(B) UMAP visualization showing ESRRG expression in all cells from treatment-naive samples and chemotherapy-treated samples.

(C) AUC score of ECM pathway in treatment-naive samples and chemotherapy-treated samples.

(D) Body weight of mice bearing LN140 PDX tumors were treated with vehicle, DN200434 (10 mg/kg/day, ip) and Chemo (Day 1, DDP 2.5 mg/kg, ip; Day1-3, Eto 4 mg/kg, ip) alone or combination (n = 6 mice per group), One week was considered as one cycle. Mean mice body weight ± s.e.m. was shown. Significance was calculated using Student's t-test. n = 5. ns, not significant.

(E) Representative ERRγ immunostaining of tumor sections in above Figure (D) on the final day. Scale bar, 200 μm (n = 3 biological replicates).

(F) Representative IHC statistical result of indicated tumor sections from mice as in Figure (E) (n = 3 biological replicates).

(G) Representative Ki67 immunostaining and C-Caspase3 immunostaining of tumor sections in above Figure (D) on the final day (n = 3 biological replicates).

Data information: Data represent different numbers (n) of biological replicates. Data shown in (F-G) are presented as mean ± s.d. Data shown in (D) are presented as mean ± s.e.m. Student's t-test is used in (D, F, G). *$p < 0.05$, **$p < 0.01$, ***$p < 0.001$, ****$p < 0.0001$. ns, not significant. Source data are available online for this figure.

Appendix Table S1. Chemicals

Chemicals	Vendor	Catalogue number
DN200434	WuXI AppTec	EW24210-2-P1
Etoposide	Acmec	E39331135
cis-platinum	MACKLIN	15663-27-1
doxorubicin	TargetMol	T1020

Appendix Table S2. Primers for qPCR used in this study

Gene	Primers
β-actin	F：GAGAAAATCTGGCACCACACC<br>R：ATACCCCTCGTAGATGGGCAC
ESRRG	F: CTGGTAAAGAAATACAAGAGCATGAAGC<br>R: CAGCATCTTGCCAGCTCGACGAGGGTCT
COL6A1	F: ATTGCCAAGGACTTCGTCGT<br>R: TCCACTGCAGGCTCTTGATG
PIK3R2	F: CTAGCAAGATCCAGGGCGAG<br>R: ACAACGGAGCAGAAGGTGAG
WNT4	F: CTCGTCTTCGCCGTCTTCTCA<br>R: TGGATCAGGCCCTTGAGTTTC
MMP9	F: TGTACCGCTATGGTTACACTCG<br>R: GGCAGGGACAGTTGCTTCT
ADAMTS8	F: GCTGTCTACCTGAGGAGGAAGT<br>R: TCCTCCACAGGTCCGAGAACAT
CDH15	F: GAGAACCCACTTCGGACCAG<br>R: TCTTCCGGGTCGTAGTCCTT
CNTN2	F：TCTCGCCCCAGGTCCTTT<br>R：GAAGAGGAGACAAGGGCCAC
NECTIN2	F: GAATTCCCCTACACCCCGTC<br>R: TTGCTGTAGCCGTTGCCATAT

Appendix Table S3. Antibodies for immunoblotting and immunohistochemistry

Antibody	Vendor	Catalogue number	Dilution
ERRγ	Perseus	PP-H6812-00	1:1000 (WB)
	Preoteomics		1:200 (IHC)
GAPDH	Cell signaling	#5174	1:1000
c-PARP 1	Cell signaling	9542S	1:1000
c-Caspase 7	Cell signaling	9664S	1:1000
COL6A1	Proteintech	17023-1-AP	1:1000
MMP9	Santa Cruz	sc-21733	1:1000
PIK3R2	Proteintech	67644-1-Ig	1:1000
Ki67	ZSGB-BIO	ZM-0166	——
c-Caspase 3	Cell signaling	9491S	1:100

Appendix Table S4. Primers for ChIP-qPCR used in this study

Gene	Primers
CNTN2-1	F: TTCCAGACTGTTGGTTCCTG
	R: GCTCTTTGGCACCATTCAAG
CNTN2-2	F: AAGGGGTTGATGTGTCTGTC
	R: CCAGAATGAGAGGGTCTGGA
WNT4	F: CCAGACTTACAGCCAGTTGC
	R: CAACTGGCTGTAAGTCTGG
MMP9	F: GTCAGCACTTGCCTGTCAAG
	R: CCTGTCGGTGAGATTGGTTC
NECTIN2-1	F: AGTGACAGAGTCCAACCTTG
	R: CTGGCCCATTCATTCAGGTT
NECTIN2-2	F: GCGACTTCATATCTTCTGGG
	R: CGCAGCATCCTGATTCCAAA

Appendix Table S5. Sequences for siRNA used in this study

Gene	Sequences
siCont	CAGTCGCGTTTGCGACTGG
siESRRG-1	CGGTCTCTTTCGTTTGAGGAT
siESRRG-2	CGAATGAATGTGAAATCACAA

Appendix Table S6. Sequences for shRNA used in this study

Gene	Sequences
shESRRG-1	F: CCGGCGAATGAATGTGAAATCACAACTCGAG TTGTGATTTCACATTCATTCGTTTTTG
	R: AATTCAAAAACGAATGAATGTGAAATCACAA CTCGAGTTGTGATTTCACATTCATTCG
shESRRG-2	F: CCGGCCTCACTACACTGTGTGACTTCTCGAG AAGTCACACAGTGTAGTGAGGTTTTTG
	R: AATTCAAAAACCTCACTACACTGTGTGACTT CTCGAGAAGTCACACAGTGTAGTGAGG
