# Supplementary figures and images for: Therapeutic targeting ERRγ suppresses metastasis via extracellular matrix remodeling in small cell lung cancer
Source: EMBO Mol Med. 2024 Jul 31;16(9):2043–59. doi: 10.1038/s44321-024-00108-z (PMC11393344; doi:10.1038/s44321-024-00108-z)

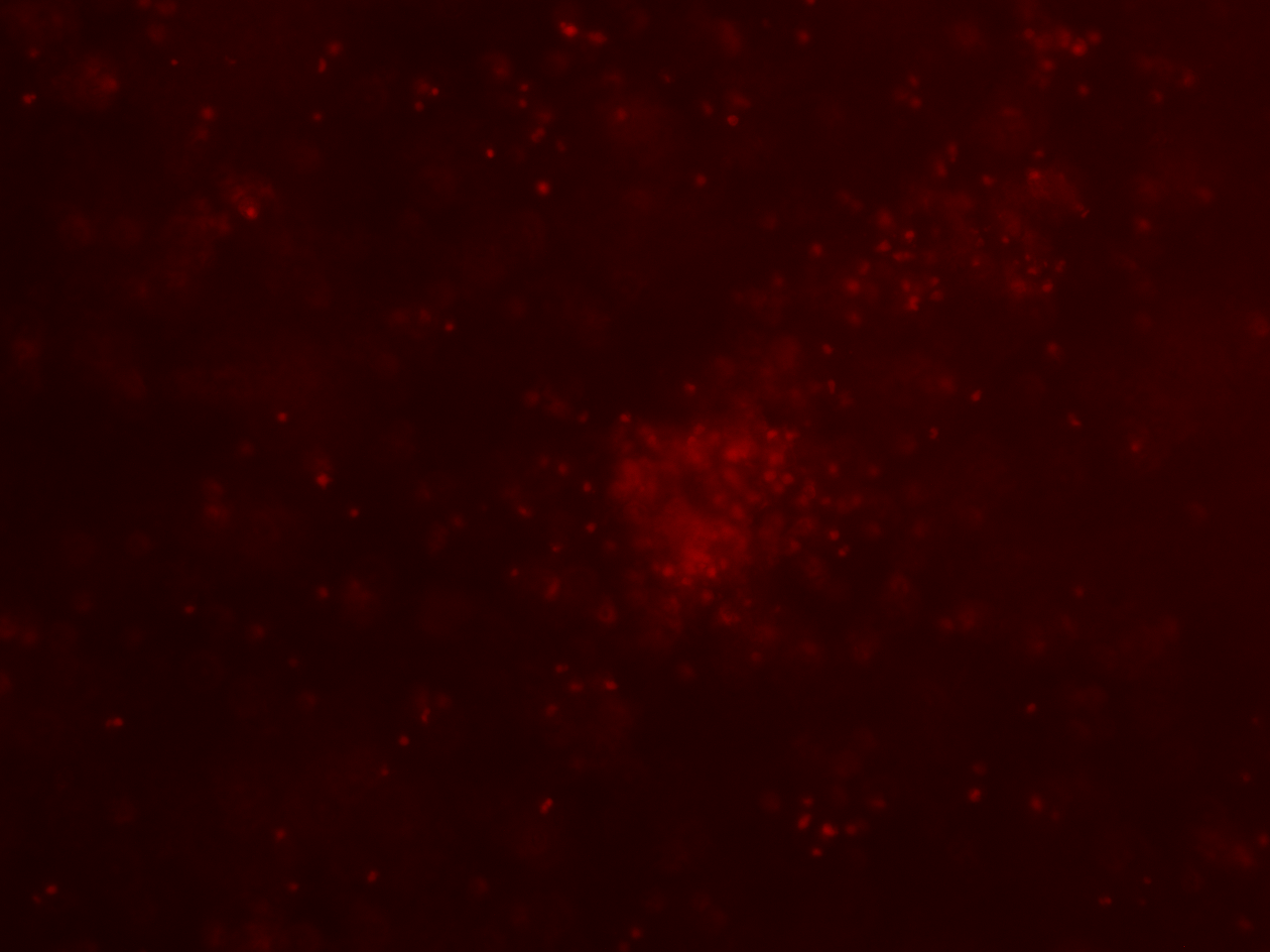

Supplement: Supplementary file 4 — Source data Fig. 2 [file 44321_2024_108_MOESM4_ESM.zip › Figure 2/2H/20-dead.tif]

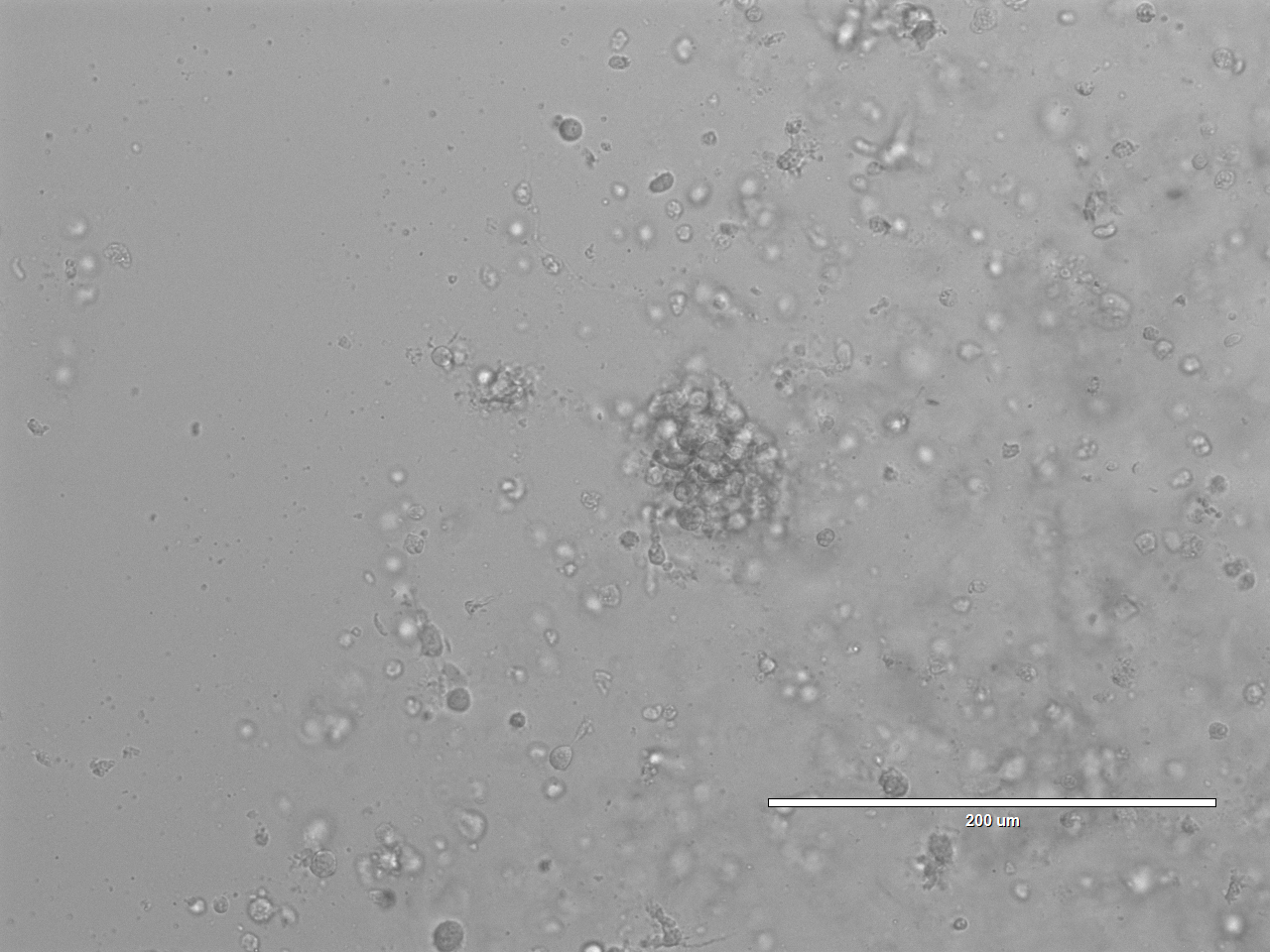

Supplement: Supplementary file 4 — Source data Fig. 2 [file 44321_2024_108_MOESM4_ESM.zip › Figure 2/2H/0-blank.tif]

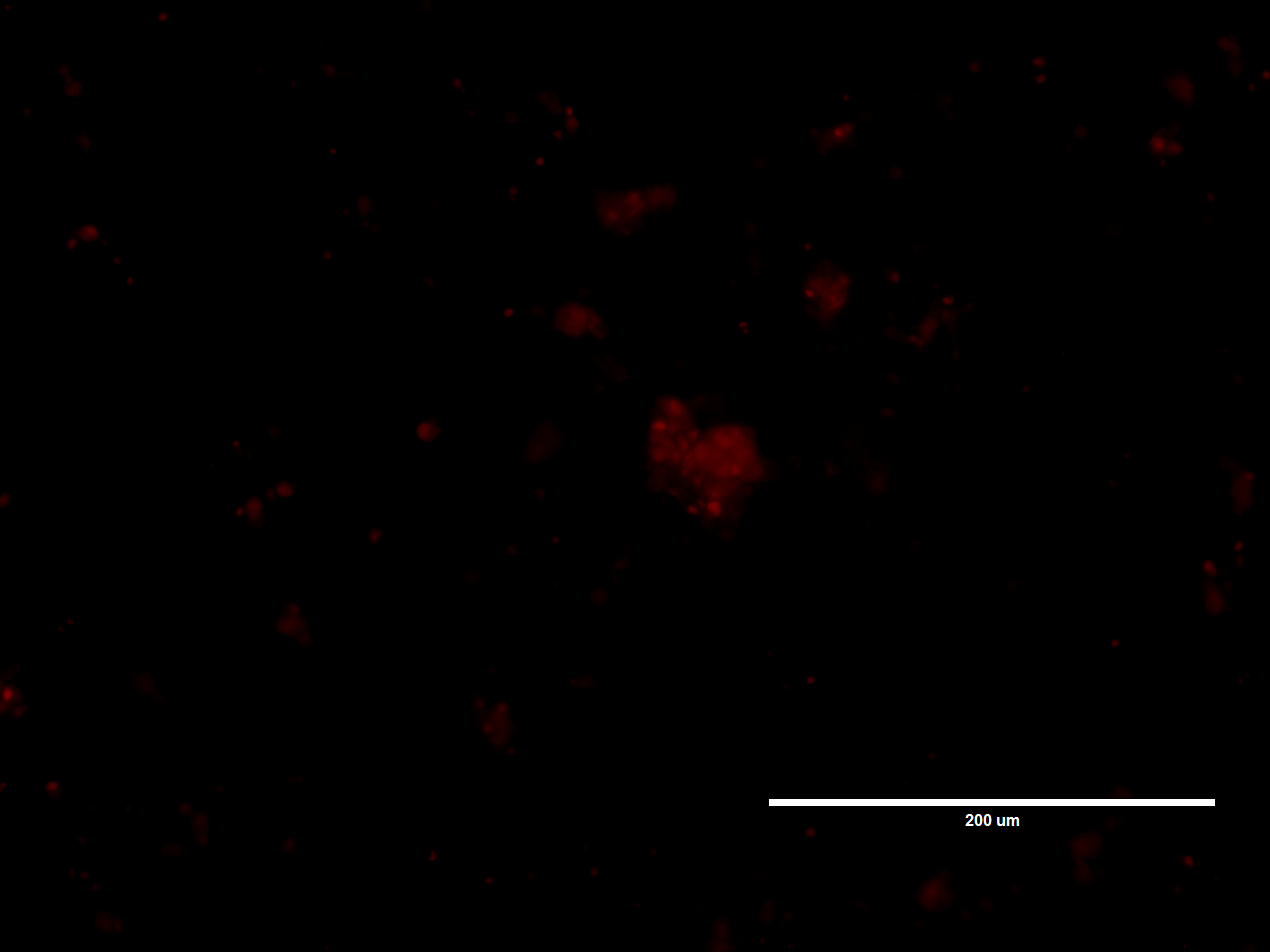

Supplement: Supplementary file 4 — Source data Fig. 2 [file 44321_2024_108_MOESM4_ESM.zip › Figure 2/2H/2.5-dead.tif]

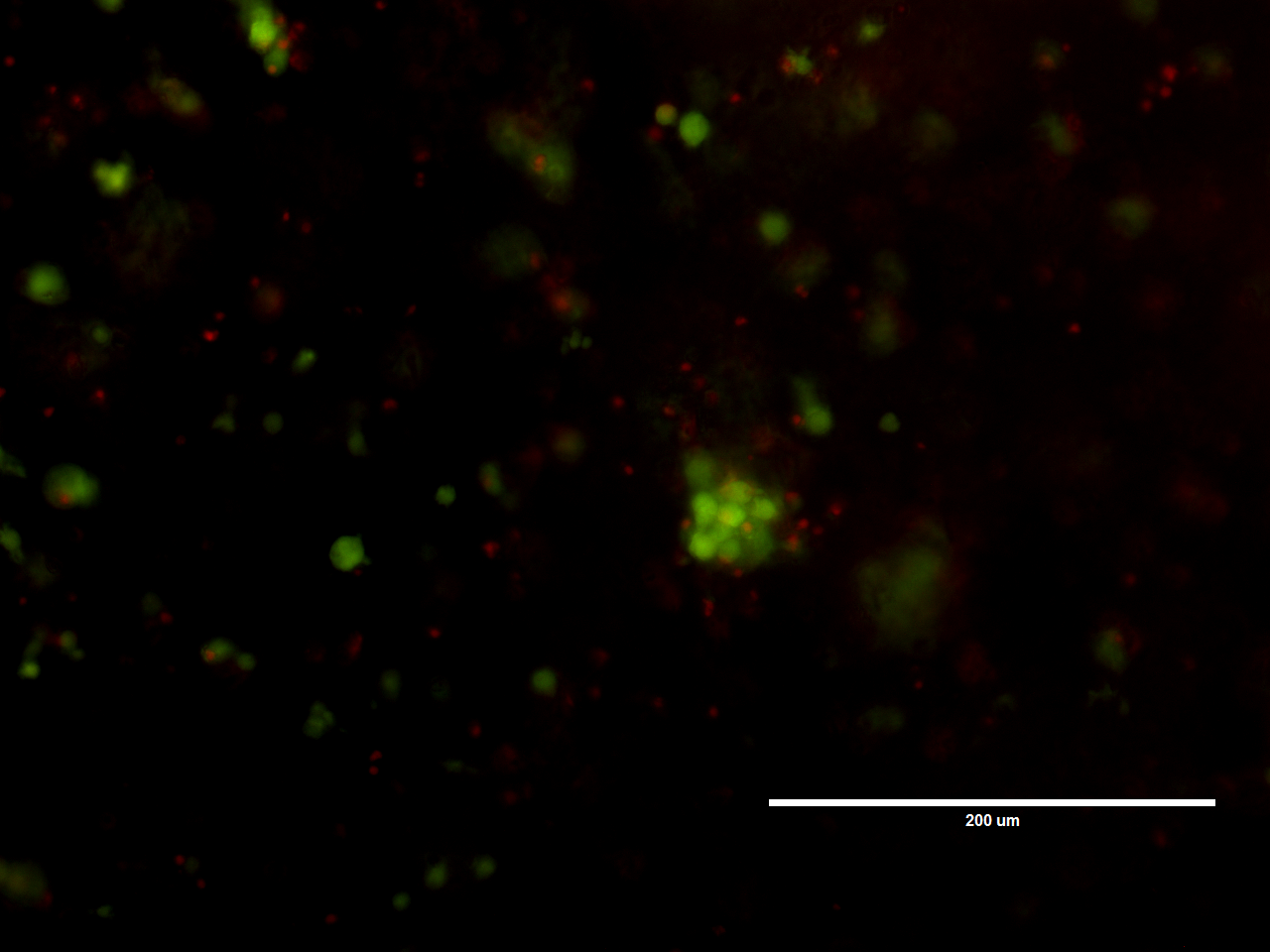

Supplement: Supplementary file 4 — Source data Fig. 2 [file 44321_2024_108_MOESM4_ESM.zip › Figure 2/2H/5-merge.tif]

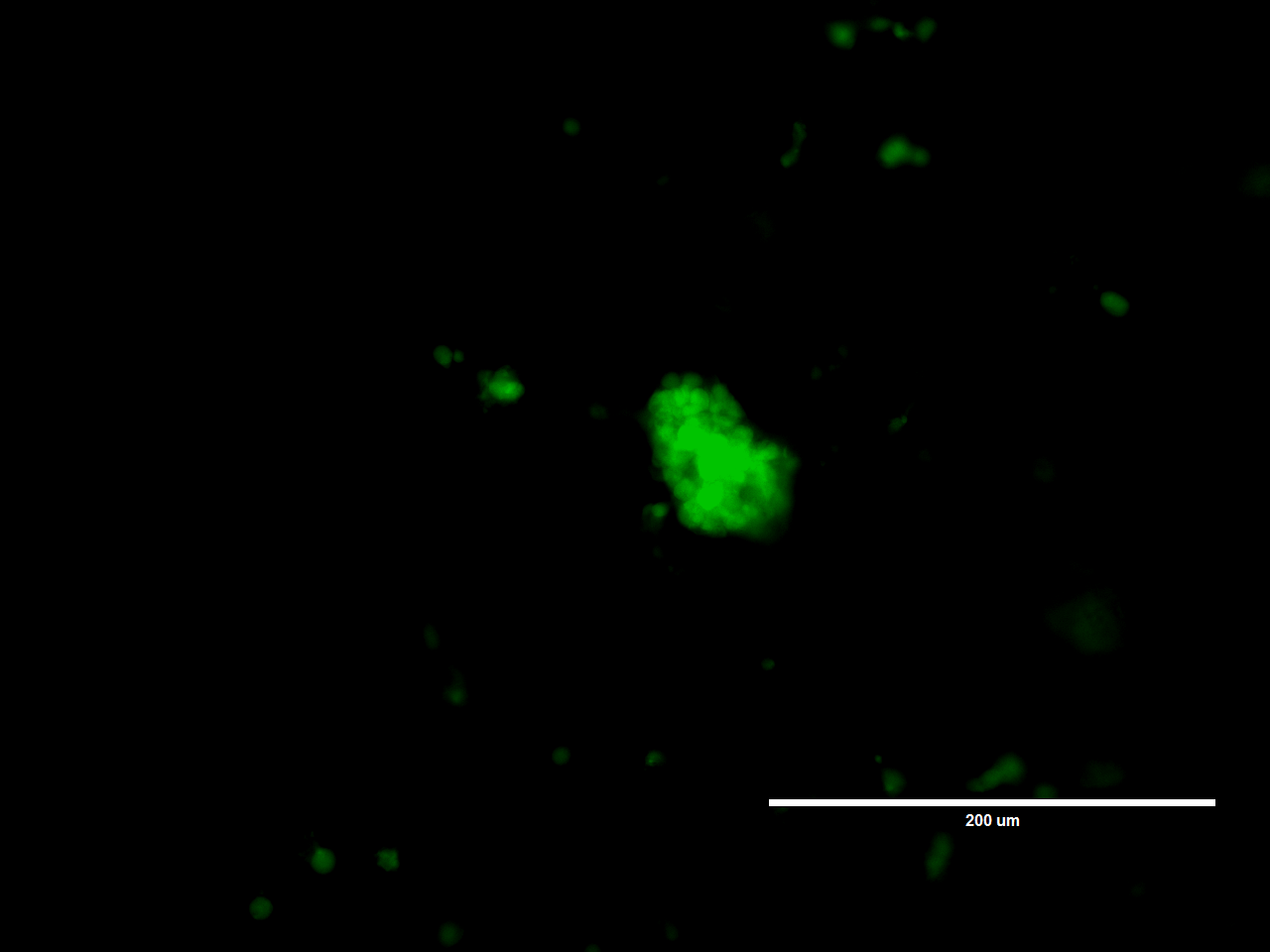

Supplement: Supplementary file 4 — Source data Fig. 2 [file 44321_2024_108_MOESM4_ESM.zip › Figure 2/2H/0-live.tif]

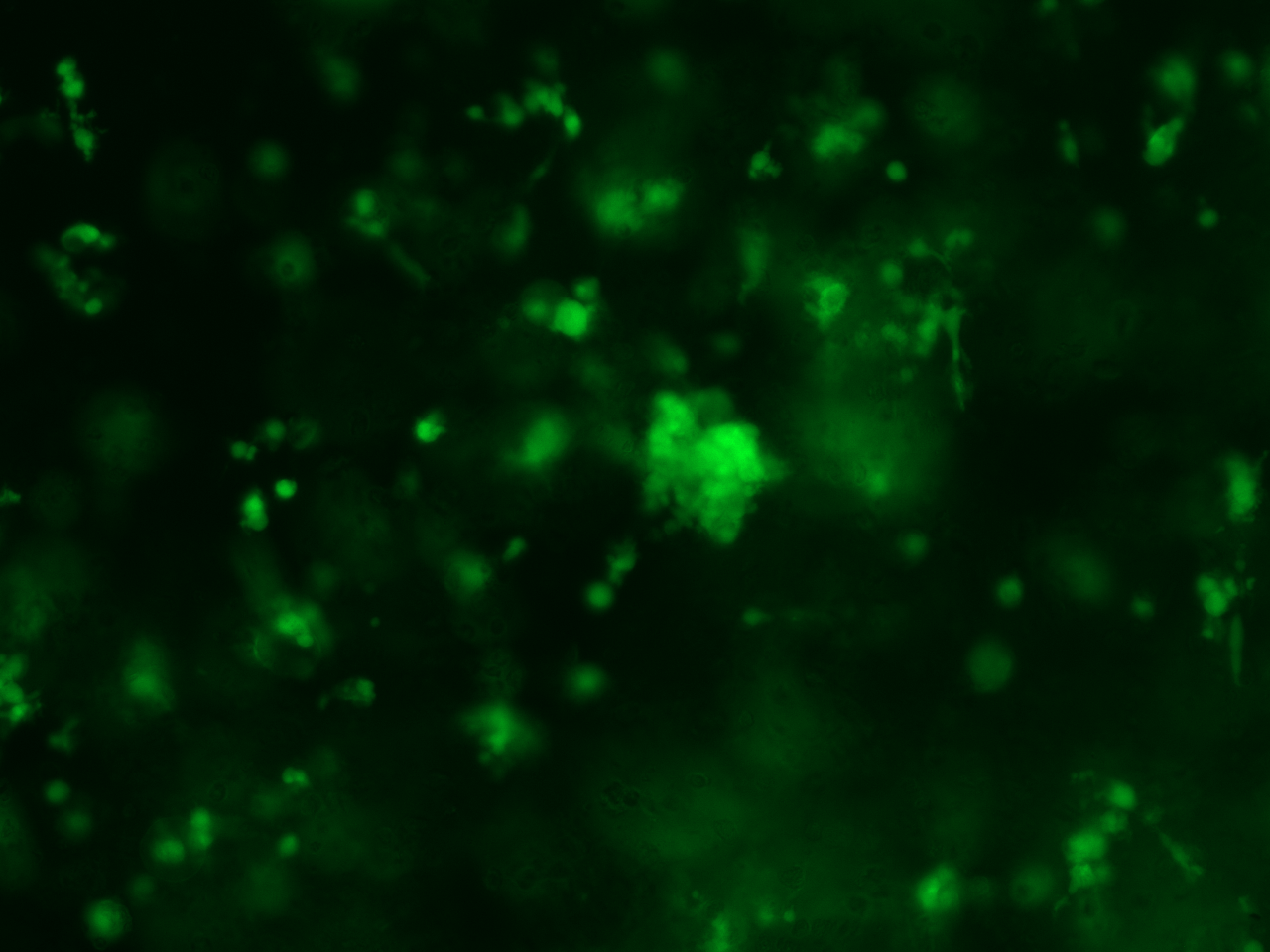

Supplement: Supplementary file 4 — Source data Fig. 2 [file 44321_2024_108_MOESM4_ESM.zip › Figure 2/2H/2.5-live.tif]

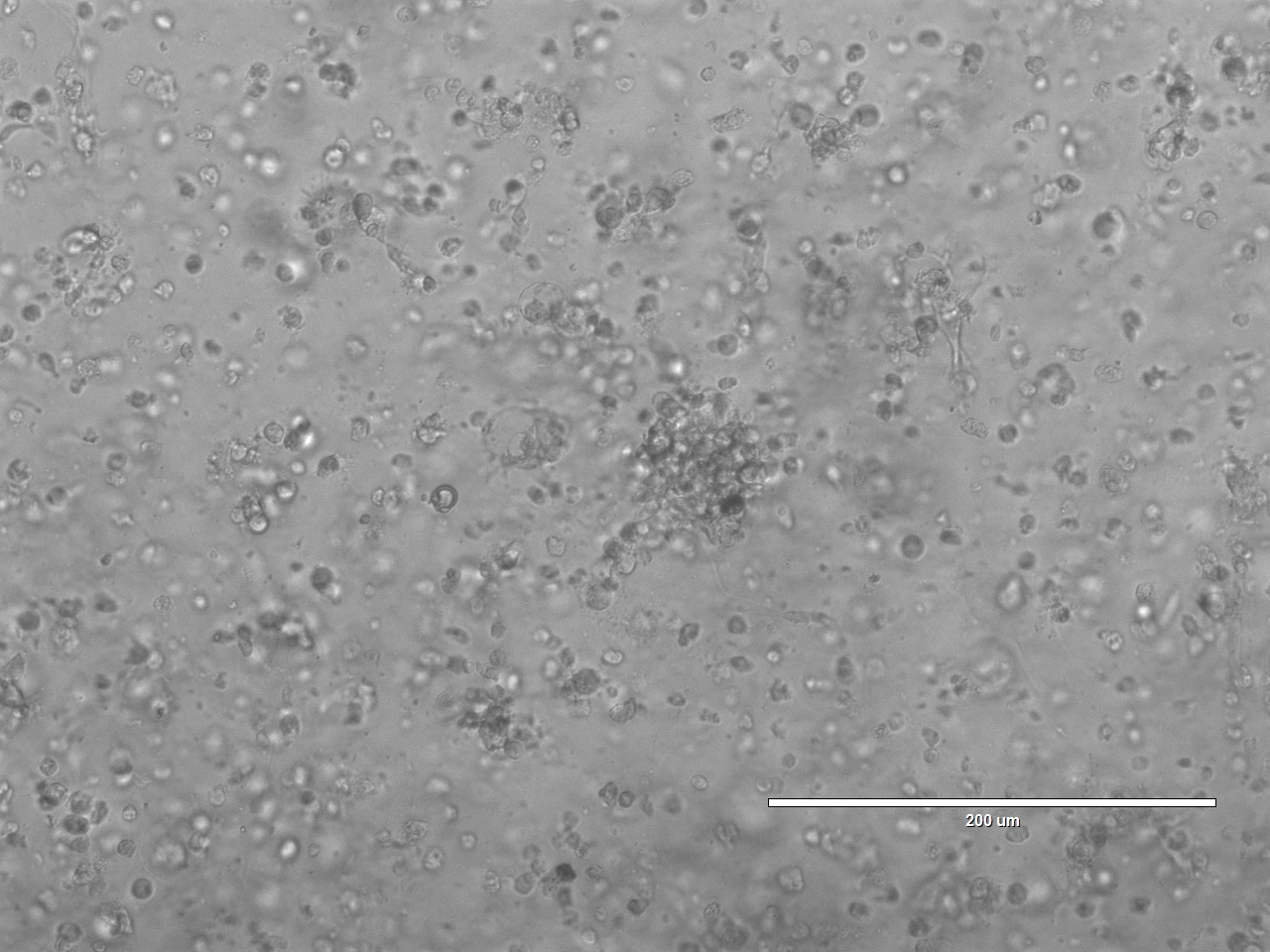

Supplement: Supplementary file 4 — Source data Fig. 2 [file 44321_2024_108_MOESM4_ESM.zip › Figure 2/2H/2.5-blank.tif]

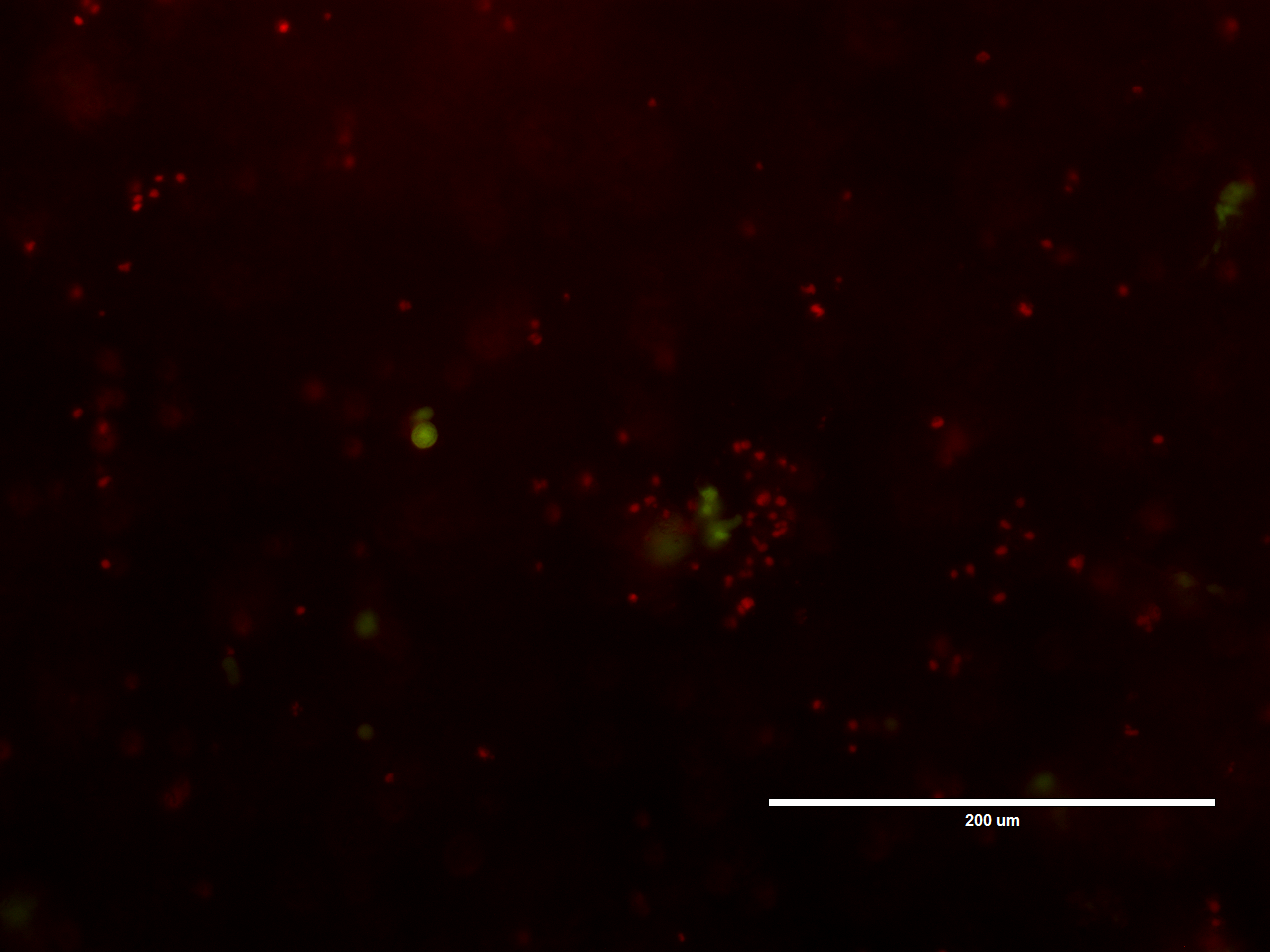

Supplement: Supplementary file 4 — Source data Fig. 2 [file 44321_2024_108_MOESM4_ESM.zip › Figure 2/2H/10-merge.tif]

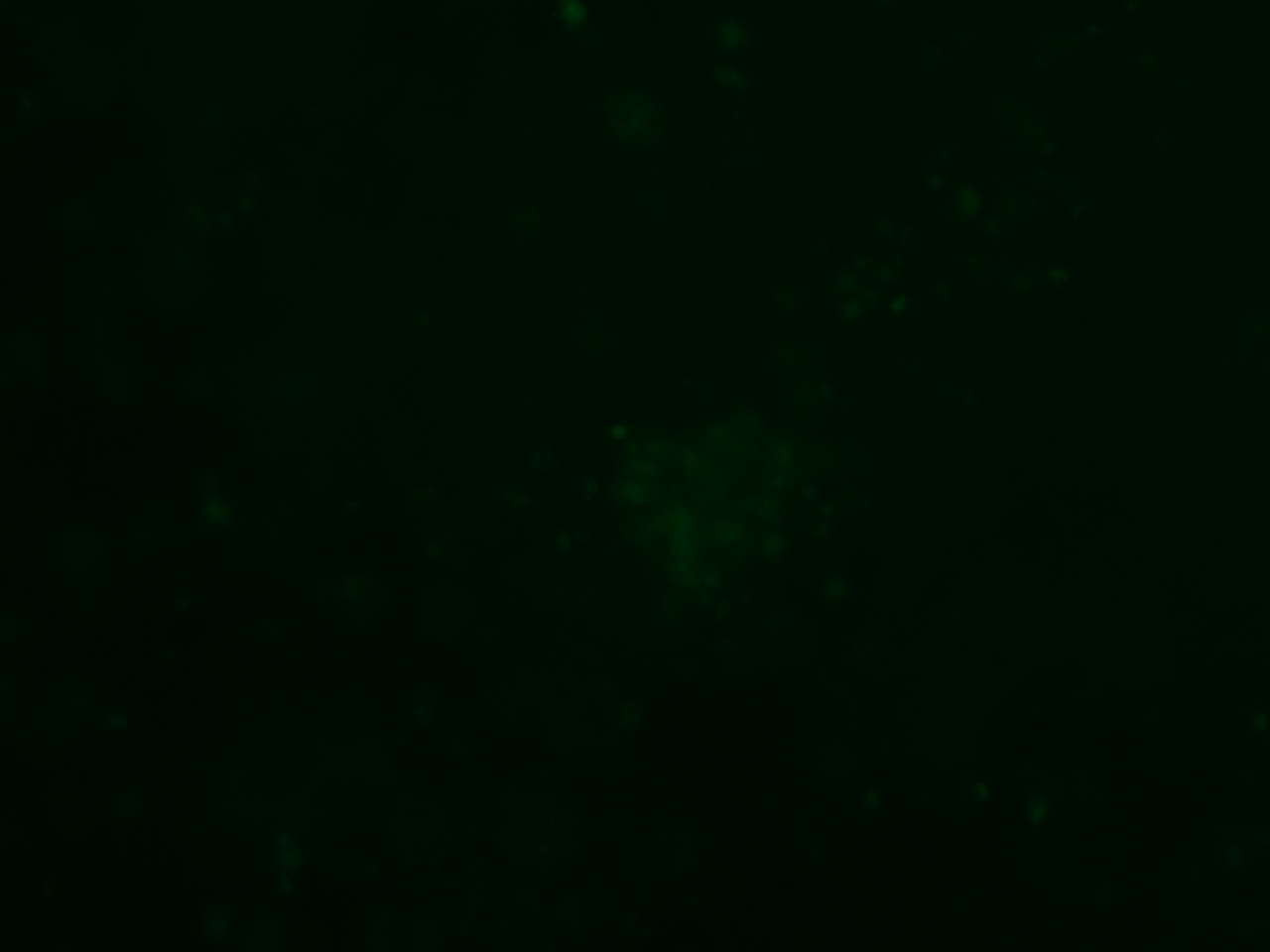

Supplement: Supplementary file 4 — Source data Fig. 2 [file 44321_2024_108_MOESM4_ESM.zip › Figure 2/2H/20-live.tif]

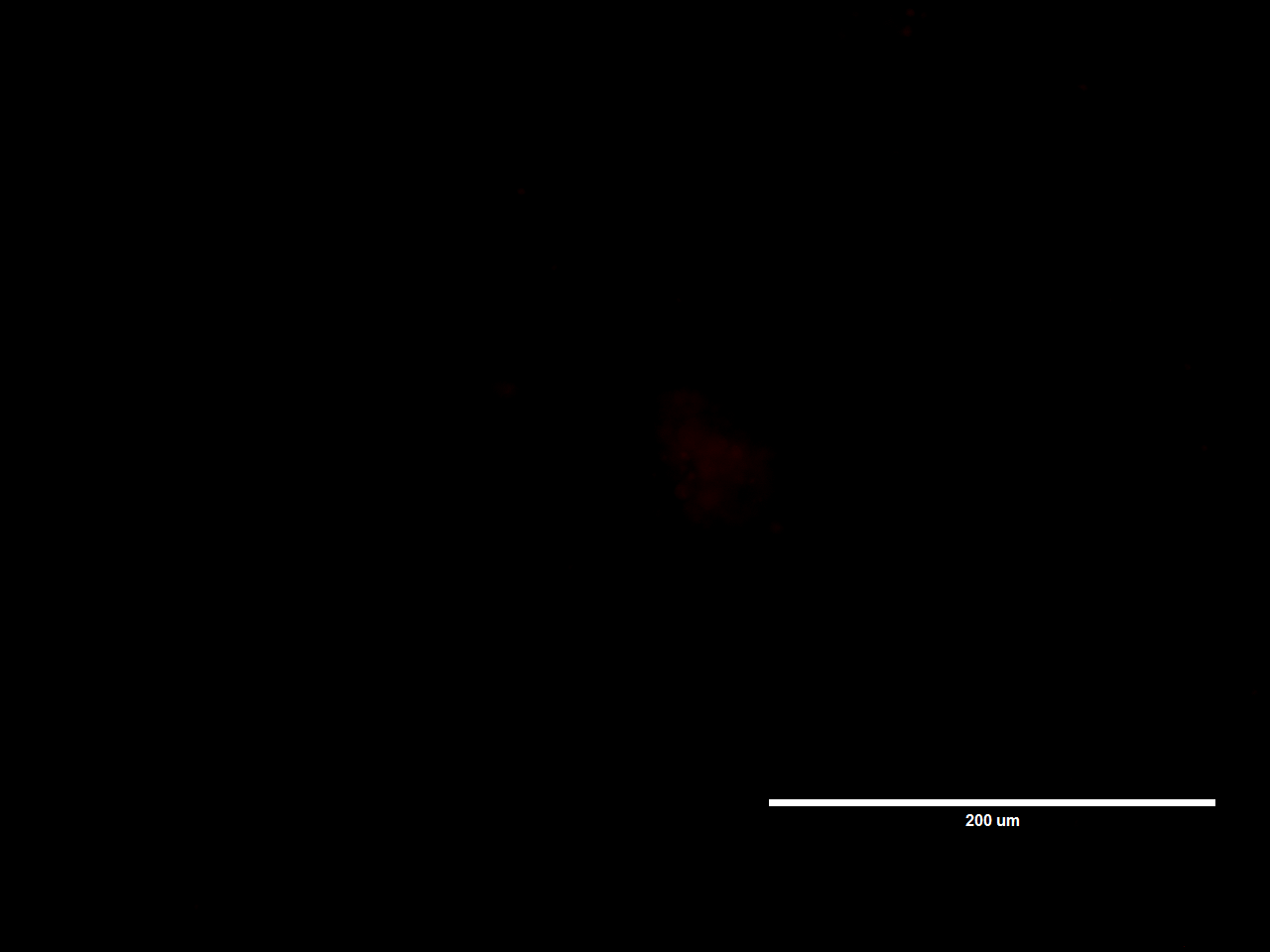

Supplement: Supplementary file 4 — Source data Fig. 2 [file 44321_2024_108_MOESM4_ESM.zip › Figure 2/2H/0-dead.tif]

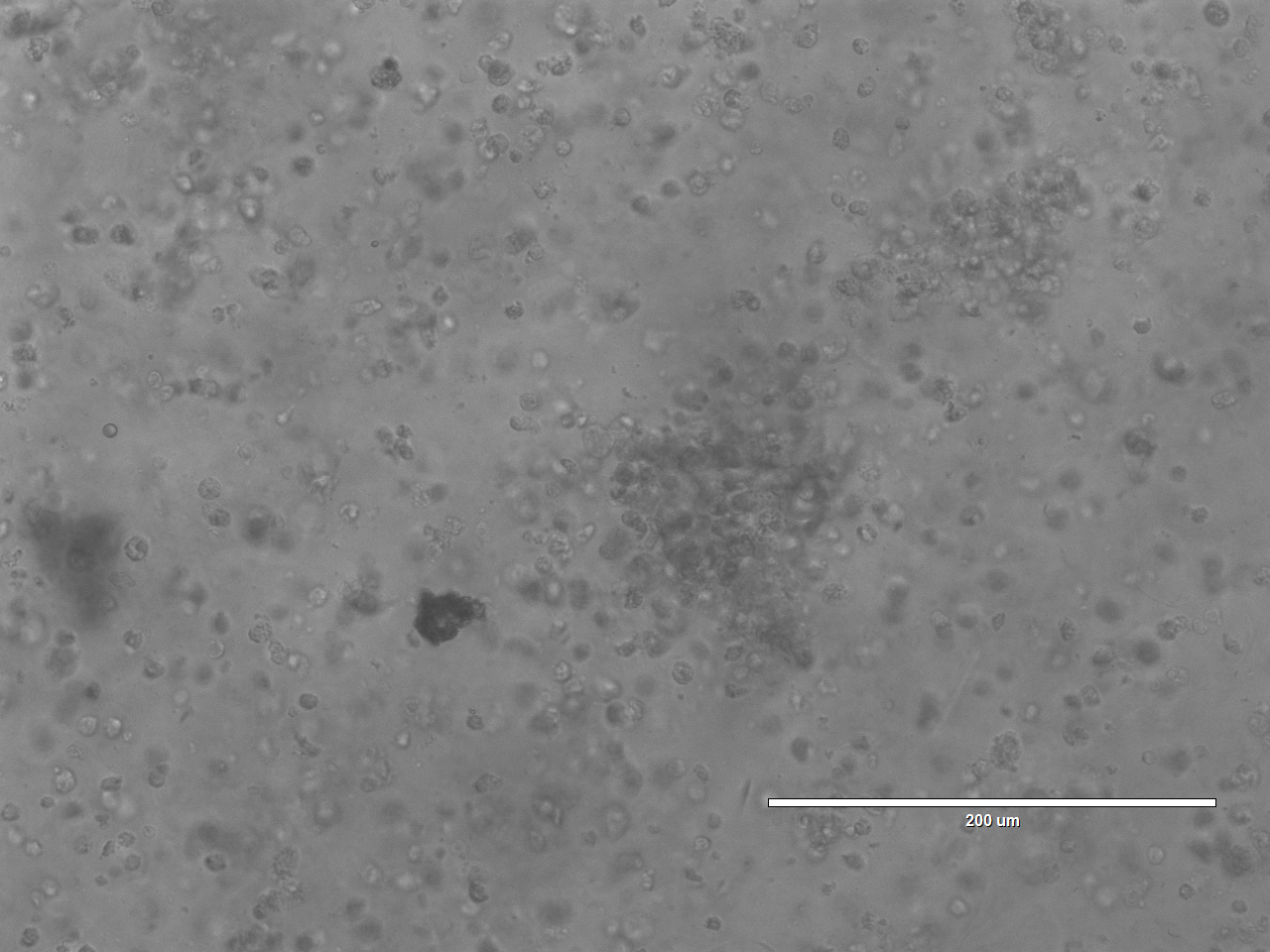

Supplement: Supplementary file 4 — Source data Fig. 2 [file 44321_2024_108_MOESM4_ESM.zip › Figure 2/2H/20-blank.tif]

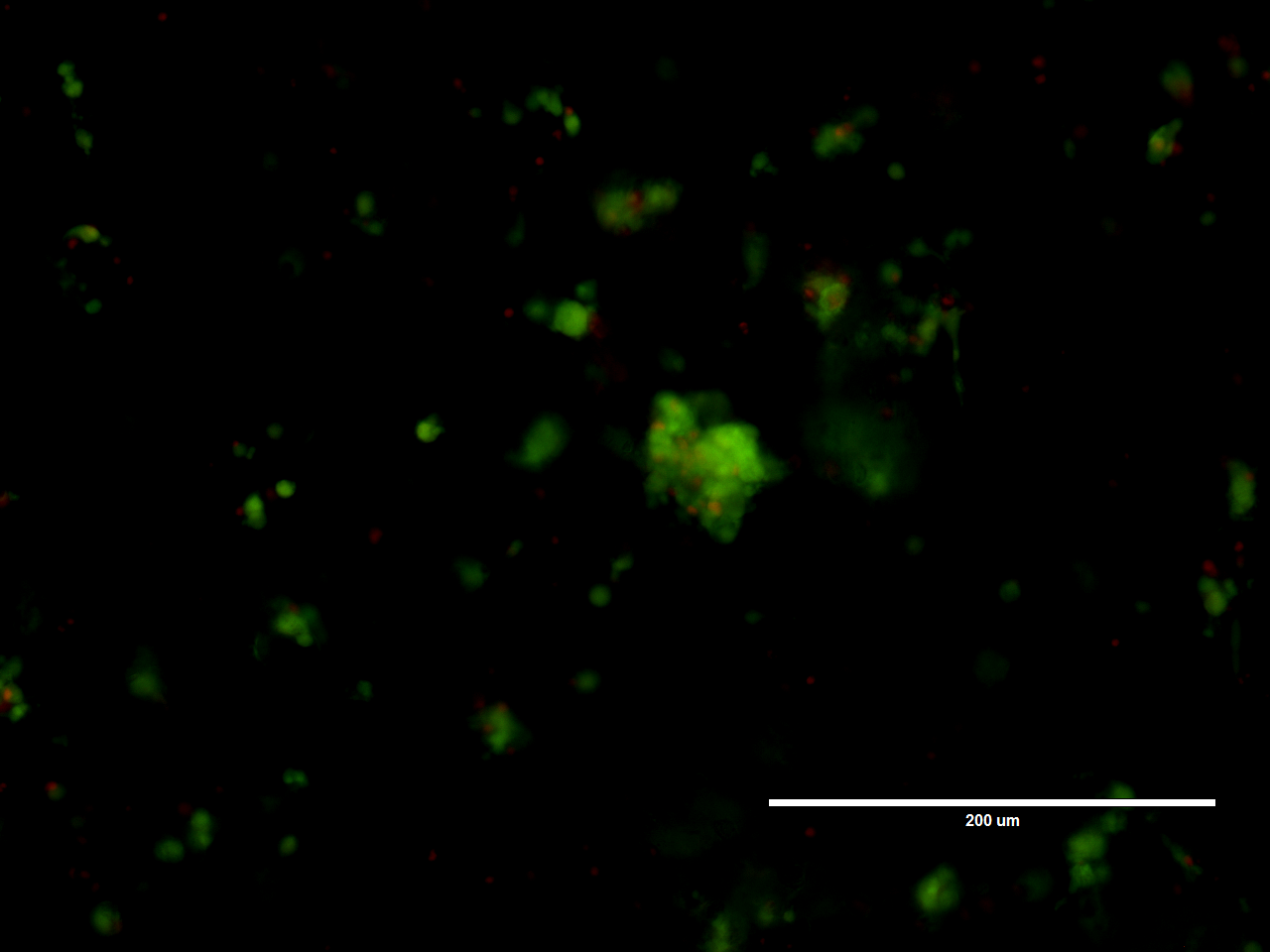

Supplement: Supplementary file 4 — Source data Fig. 2 [file 44321_2024_108_MOESM4_ESM.zip › Figure 2/2H/2.5-merge.tif]

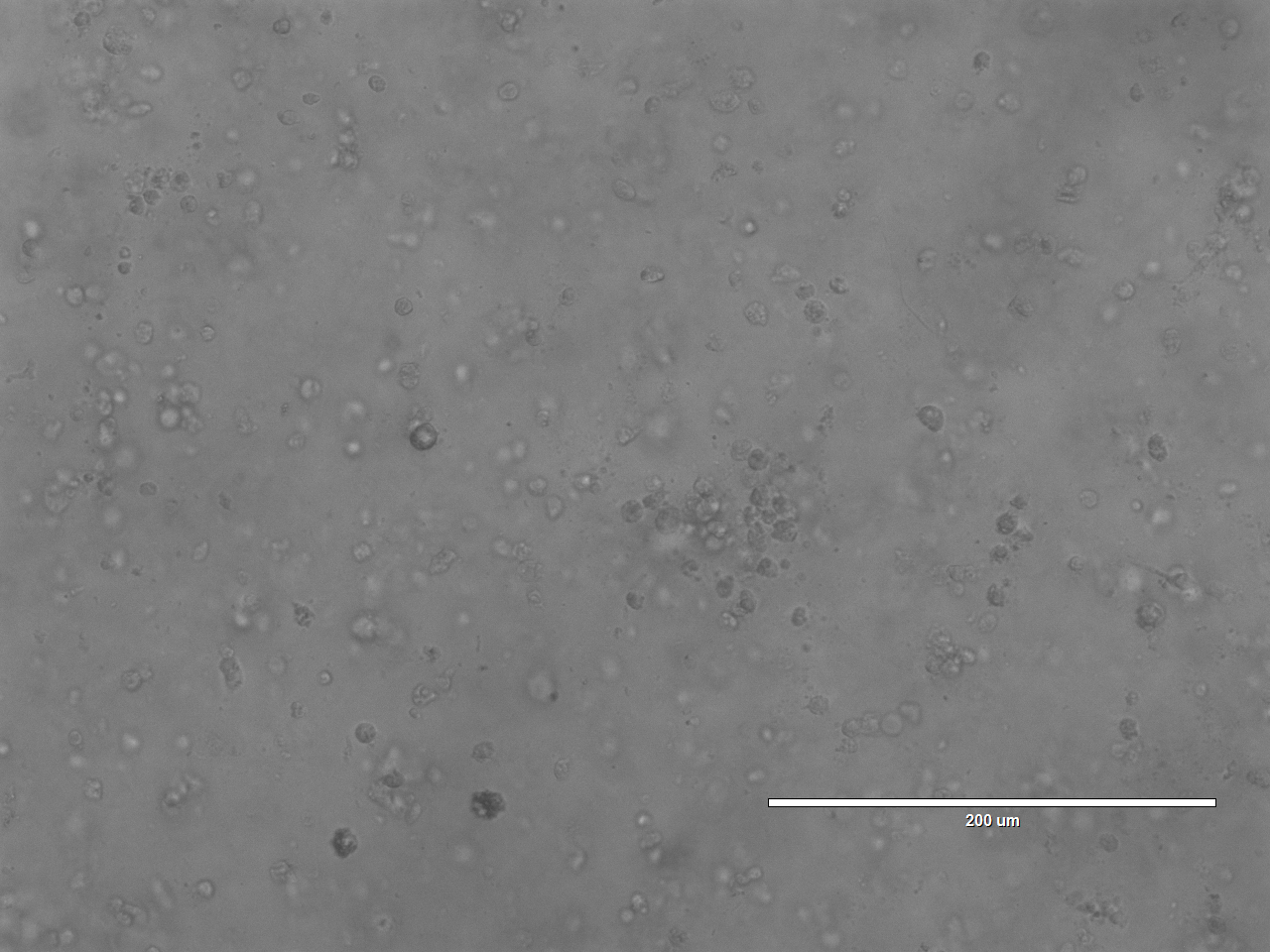

Supplement: Supplementary file 4 — Source data Fig. 2 [file 44321_2024_108_MOESM4_ESM.zip › Figure 2/2H/10-blank.tif]

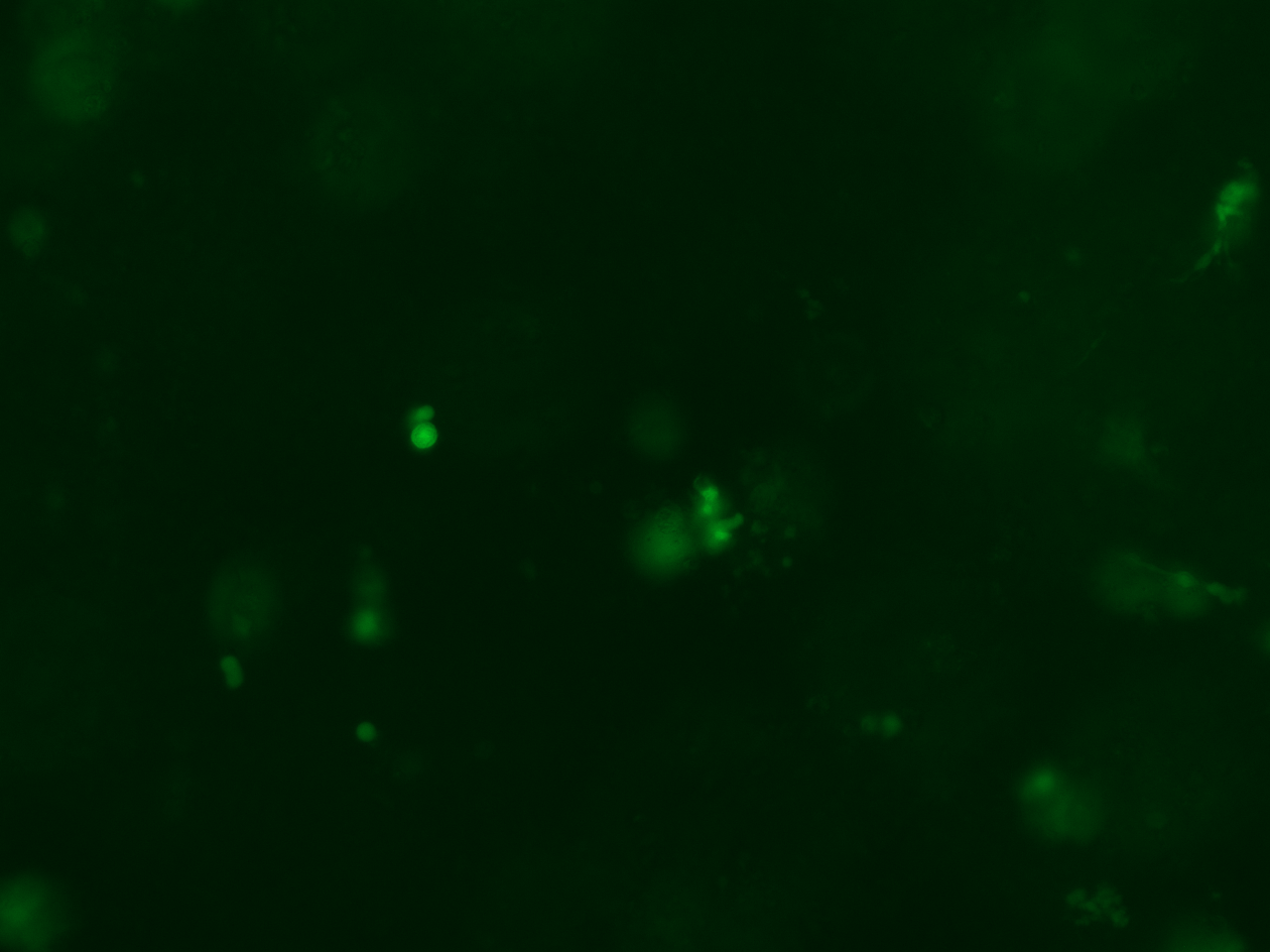

Supplement: Supplementary file 4 — Source data Fig. 2 [file 44321_2024_108_MOESM4_ESM.zip › Figure 2/2H/10-live.tif]

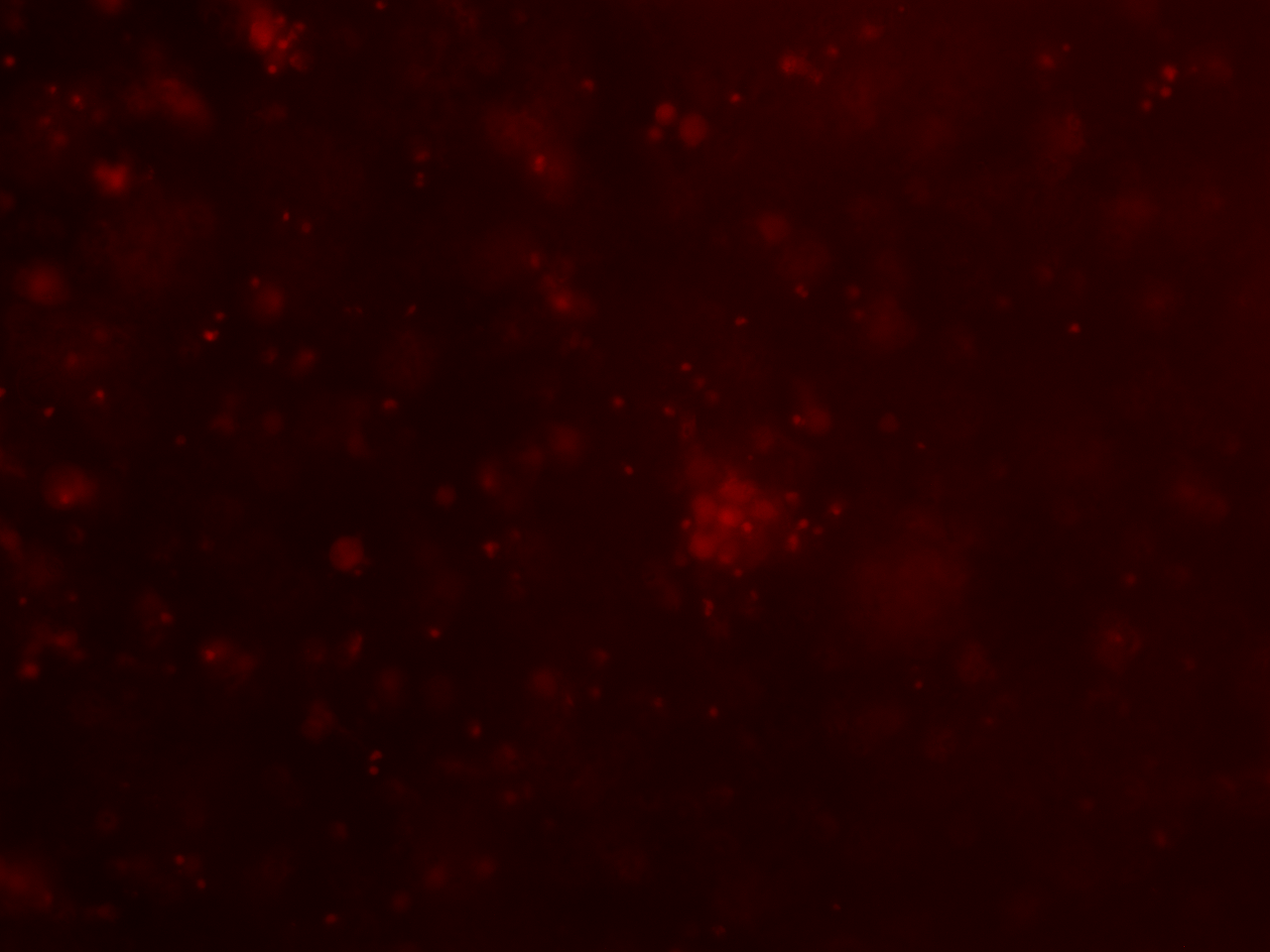

Supplement: Supplementary file 4 — Source data Fig. 2 [file 44321_2024_108_MOESM4_ESM.zip › Figure 2/2H/5-dead.tif]

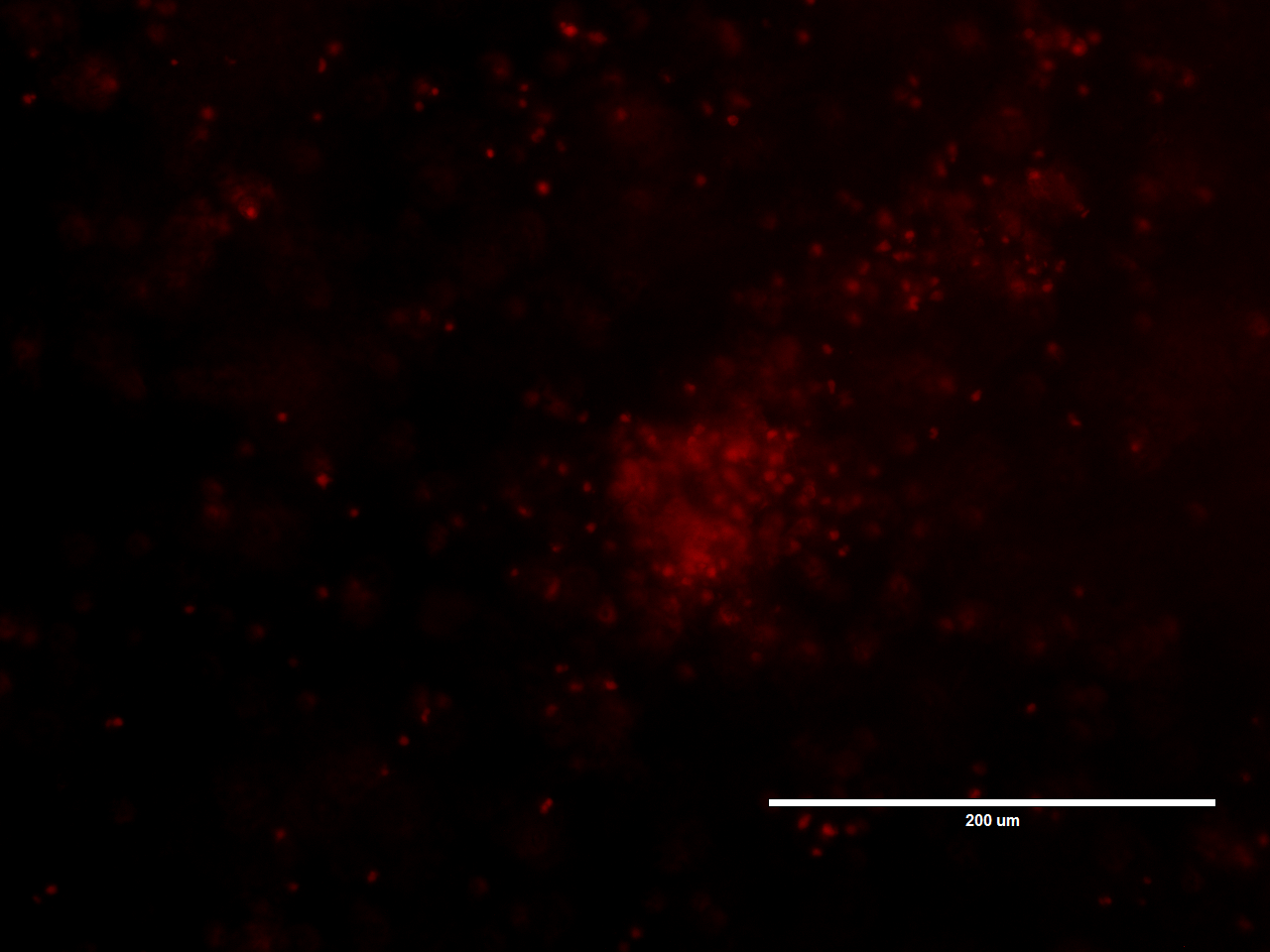

Supplement: Supplementary file 4 — Source data Fig. 2 [file 44321_2024_108_MOESM4_ESM.zip › Figure 2/2H/20-merge.tif]

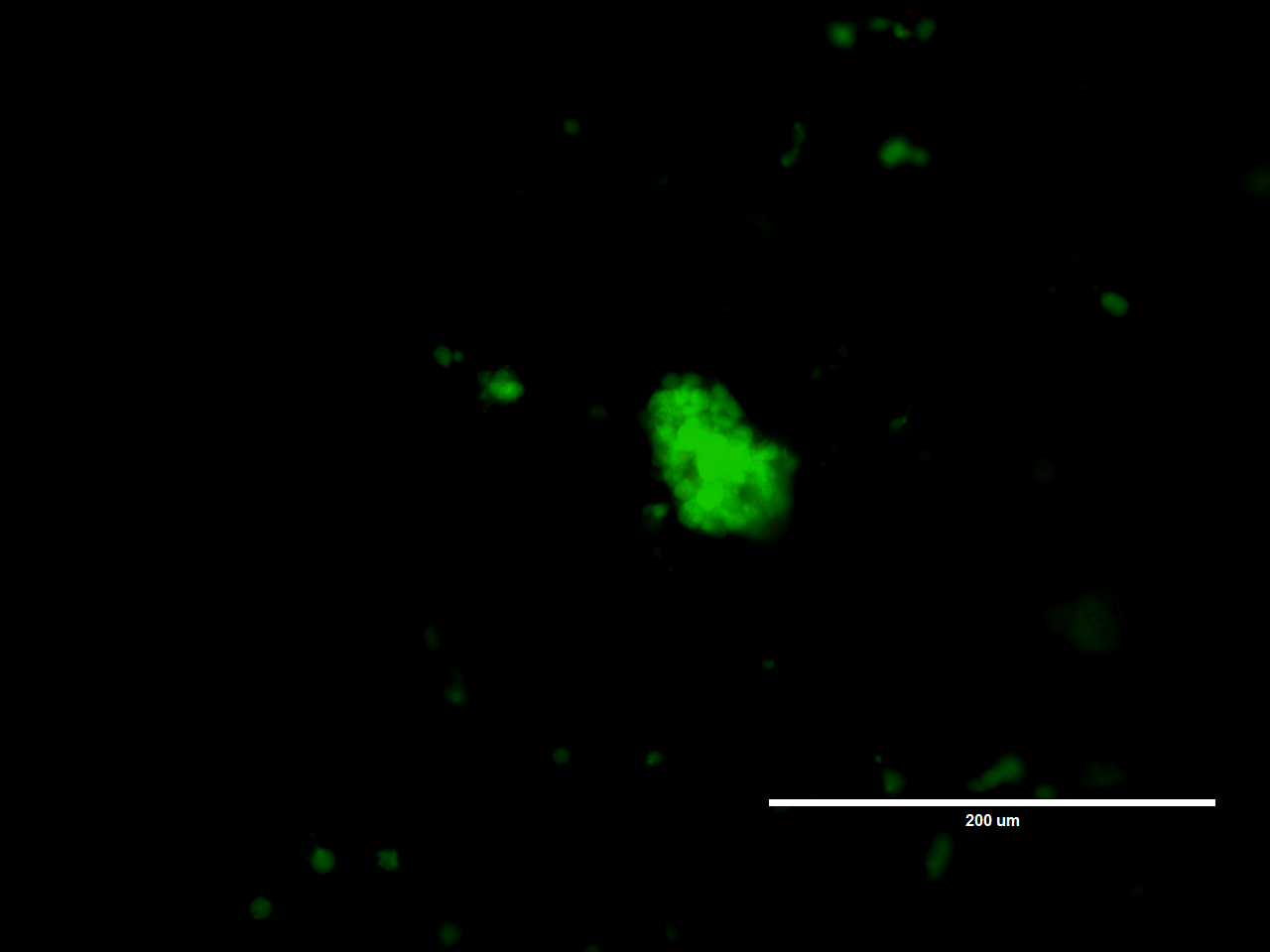

Supplement: Supplementary file 4 — Source data Fig. 2 [file 44321_2024_108_MOESM4_ESM.zip › Figure 2/2H/0-merge.tif]

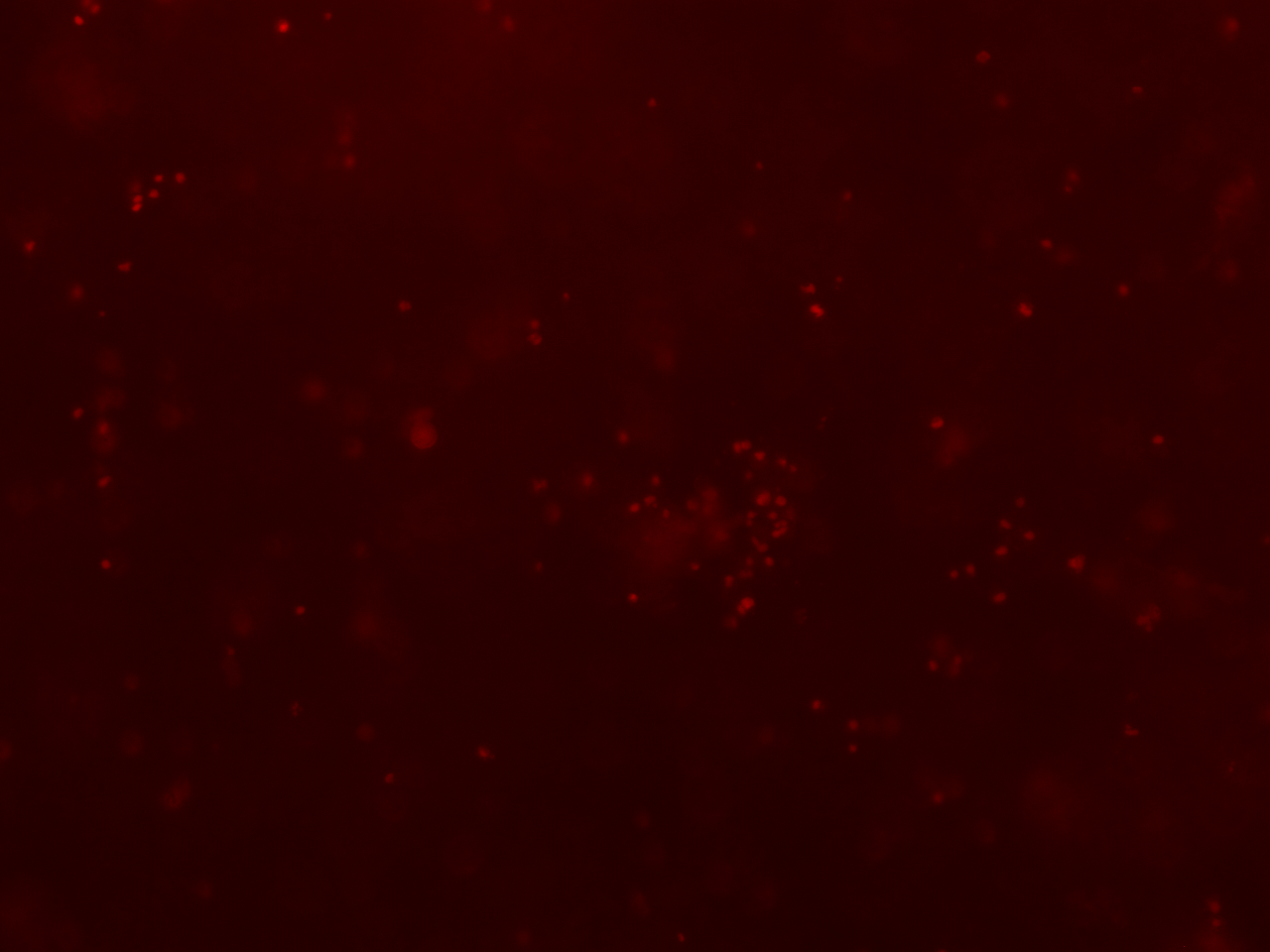

Supplement: Supplementary file 4 — Source data Fig. 2 [file 44321_2024_108_MOESM4_ESM.zip › Figure 2/2H/10-dead.tif]

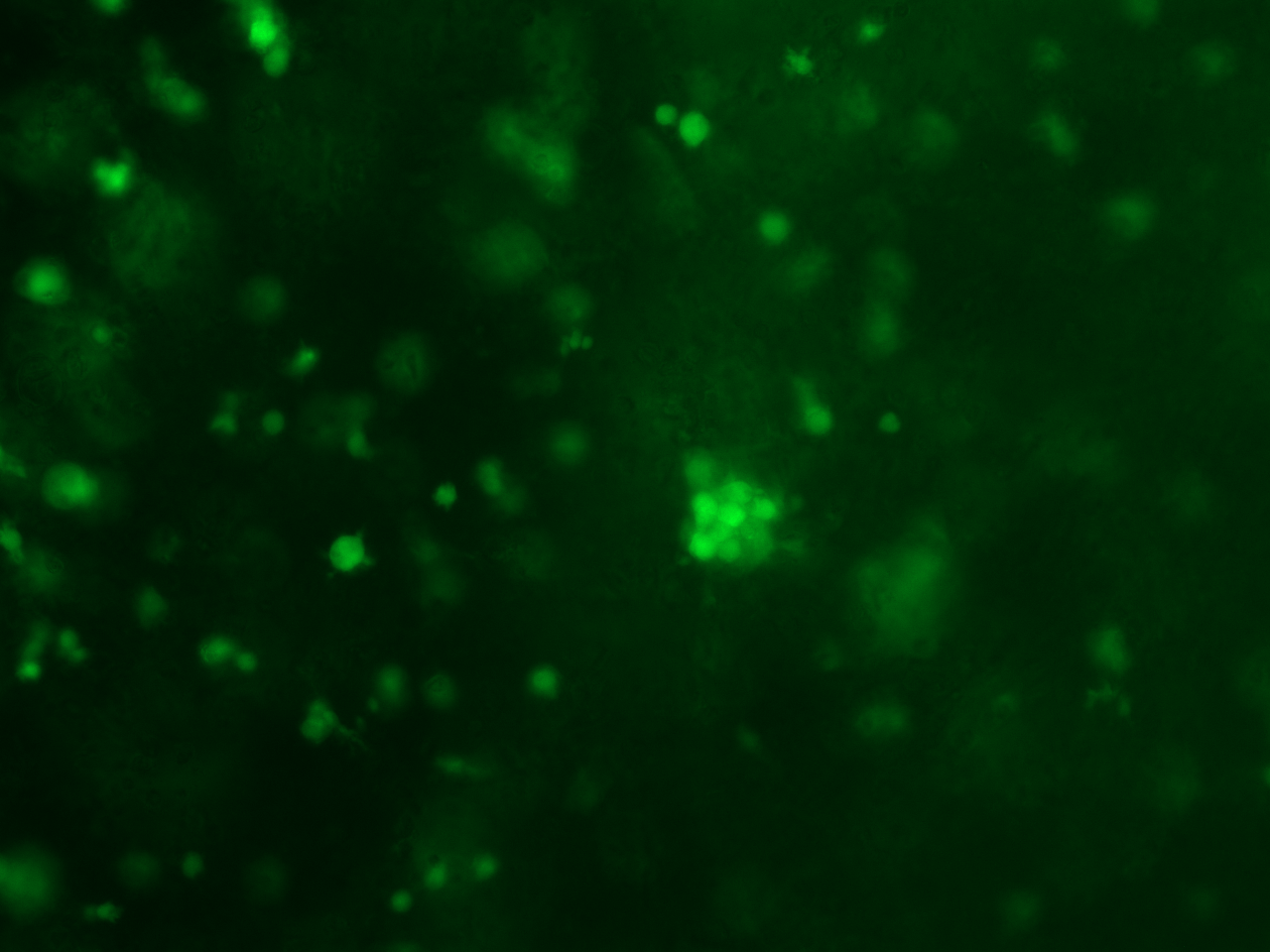

Supplement: Supplementary file 4 — Source data Fig. 2 [file 44321_2024_108_MOESM4_ESM.zip › Figure 2/2H/5-live.tif]

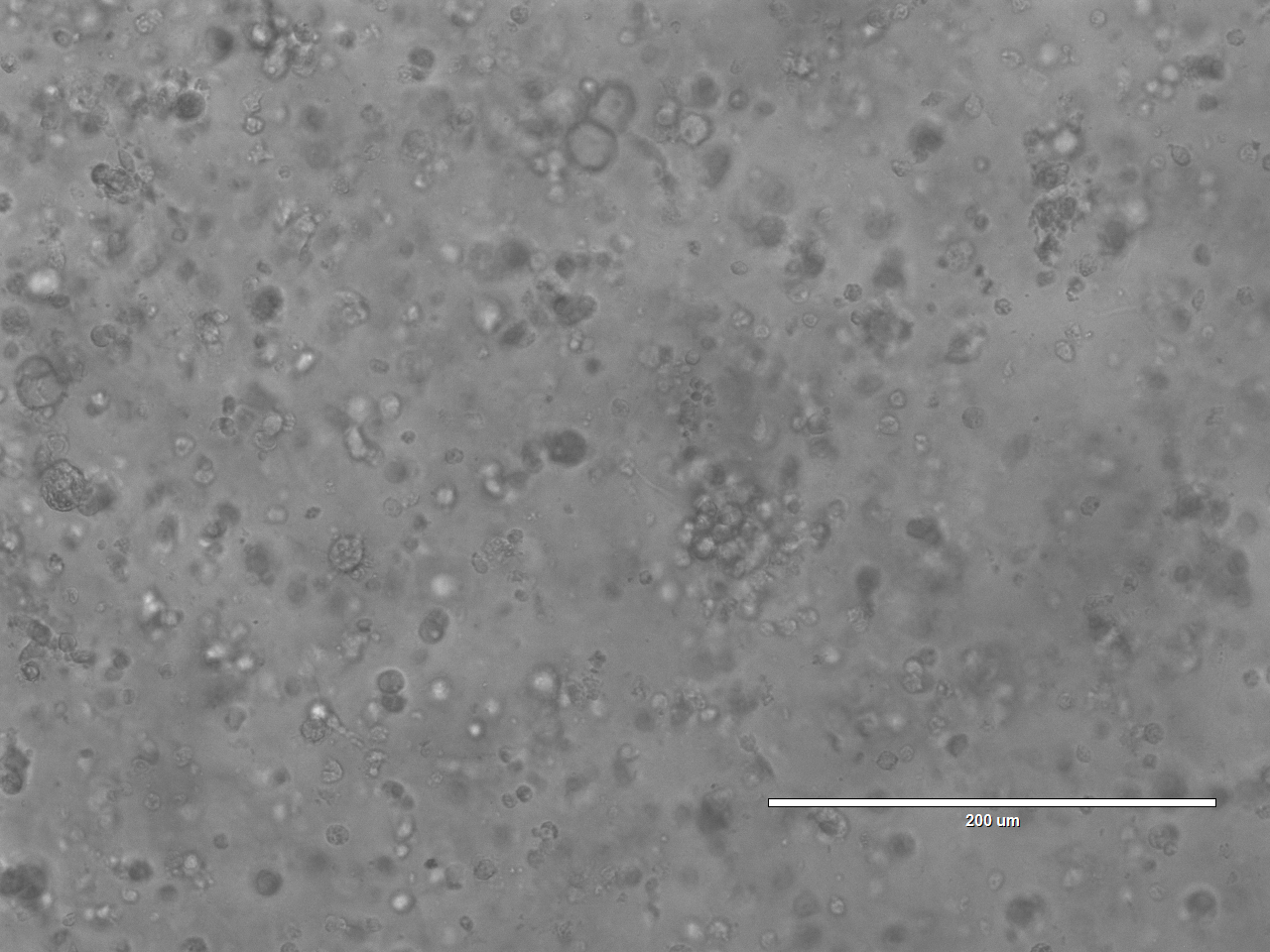

Supplement: Supplementary file 4 — Source data Fig. 2 [file 44321_2024_108_MOESM4_ESM.zip › Figure 2/2H/5-blank.tif]

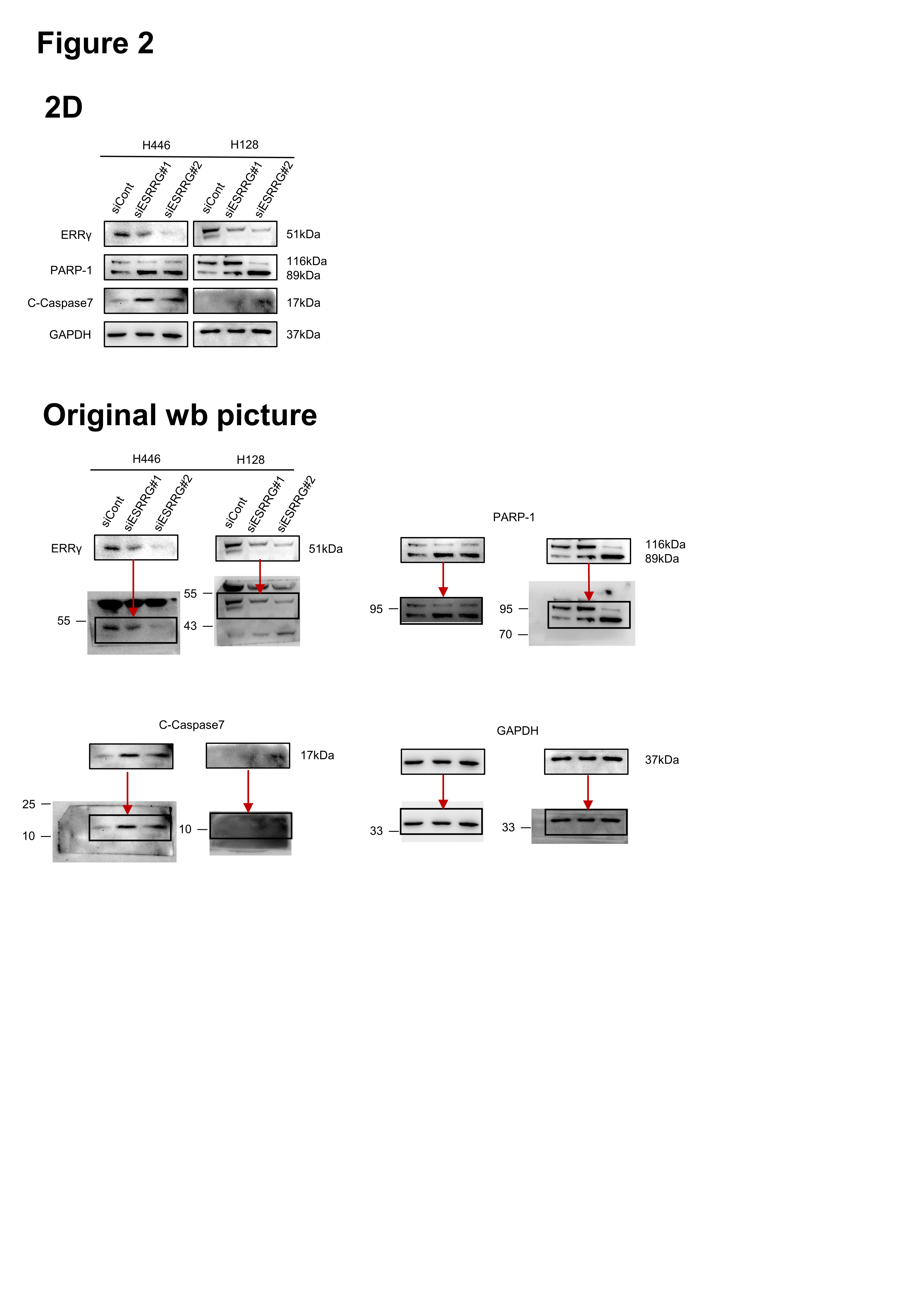

Supplement: Supplementary file 4 — Source data Fig. 2 [file 44321_2024_108_MOESM4_ESM.zip › Figure 2/2D/2D.tif]

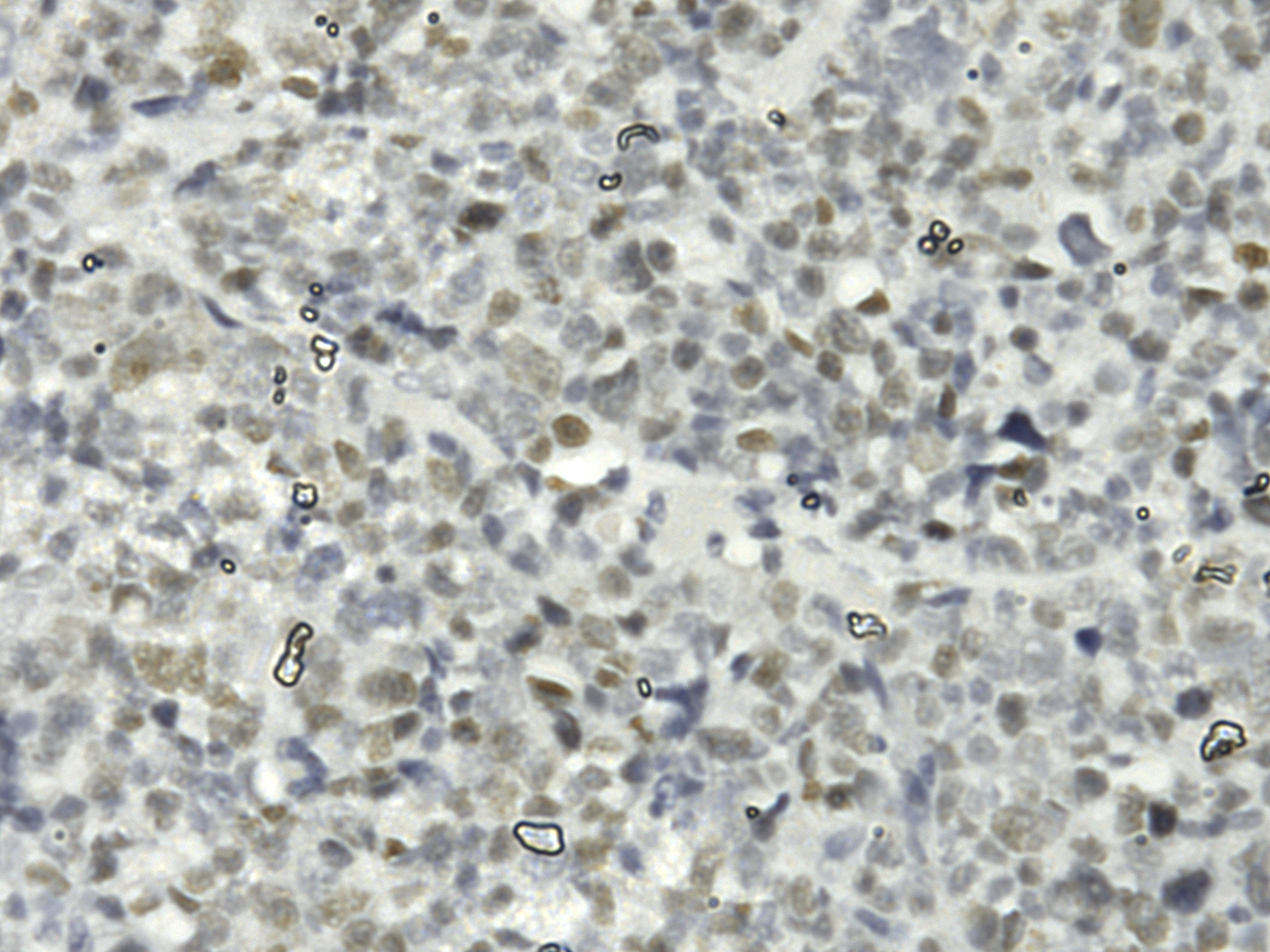

Supplement: Supplementary file 4 — Source data Fig. 2 [file 44321_2024_108_MOESM4_ESM.zip › Figure 2/2L/Ki67-control.tif]

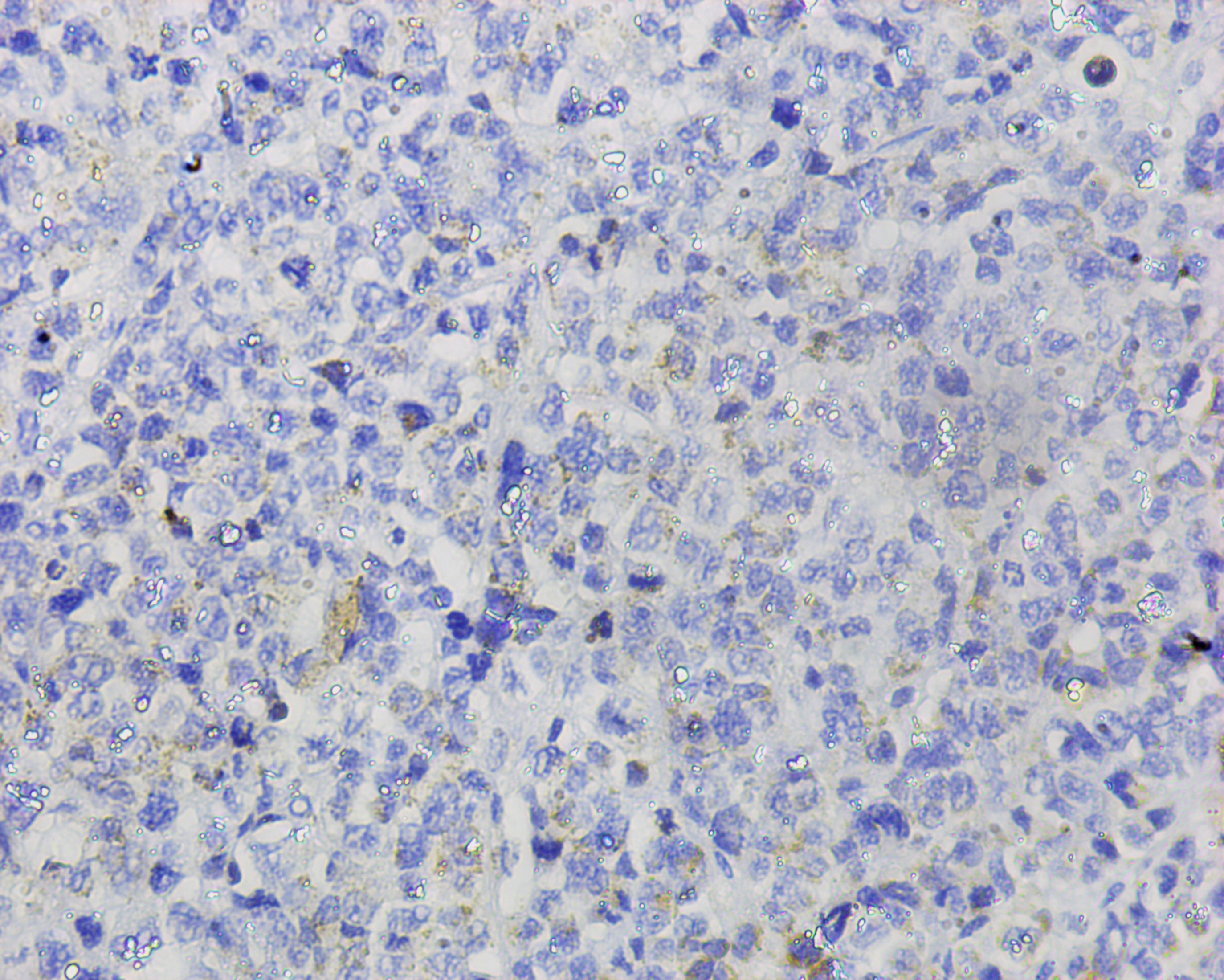

Supplement: Supplementary file 4 — Source data Fig. 2 [file 44321_2024_108_MOESM4_ESM.zip › Figure 2/2L/Con-C-Caspase3.jpg]

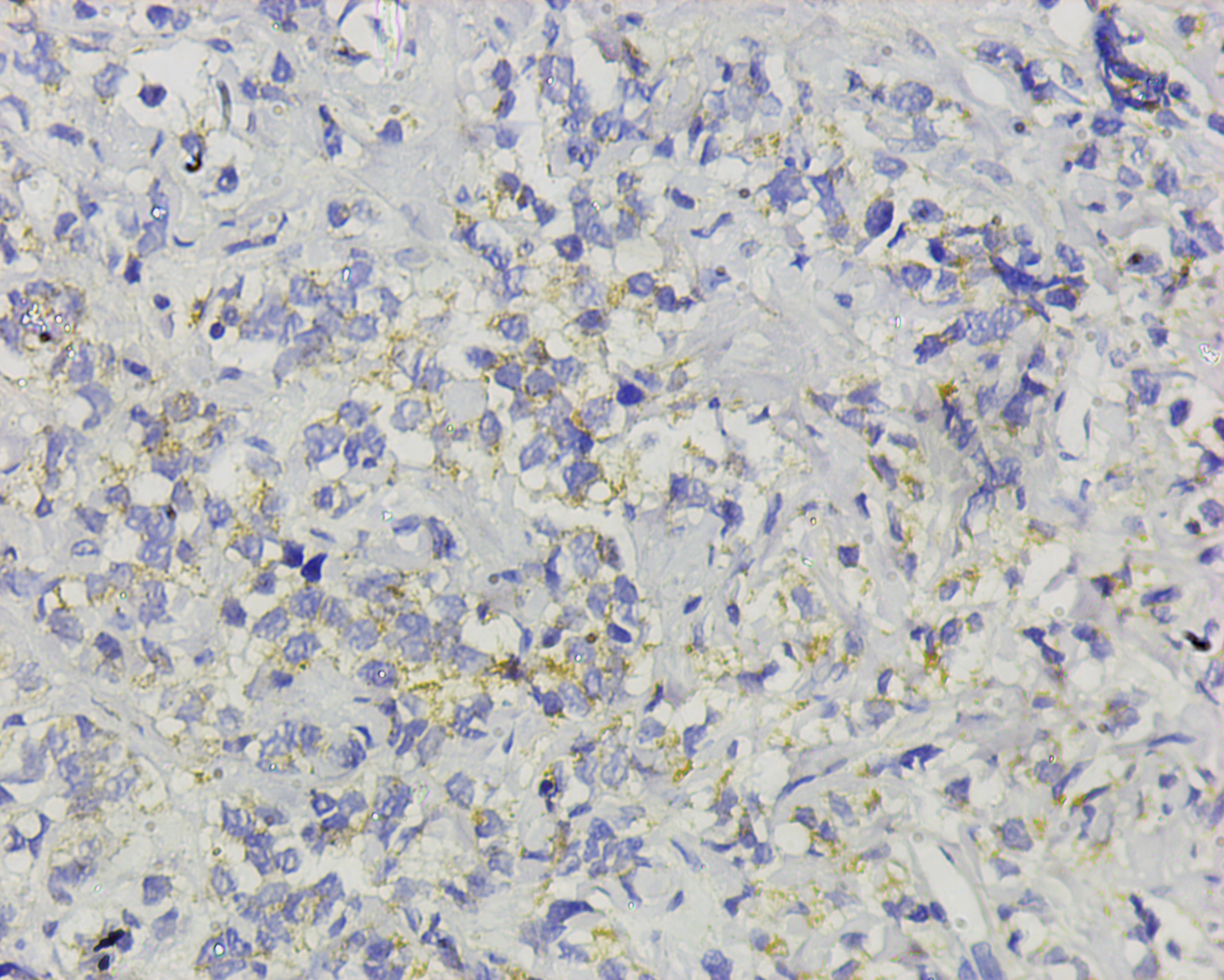

Supplement: Supplementary file 4 — Source data Fig. 2 [file 44321_2024_108_MOESM4_ESM.zip › Figure 2/2L/DN200434-C-Caspase3.jpg]

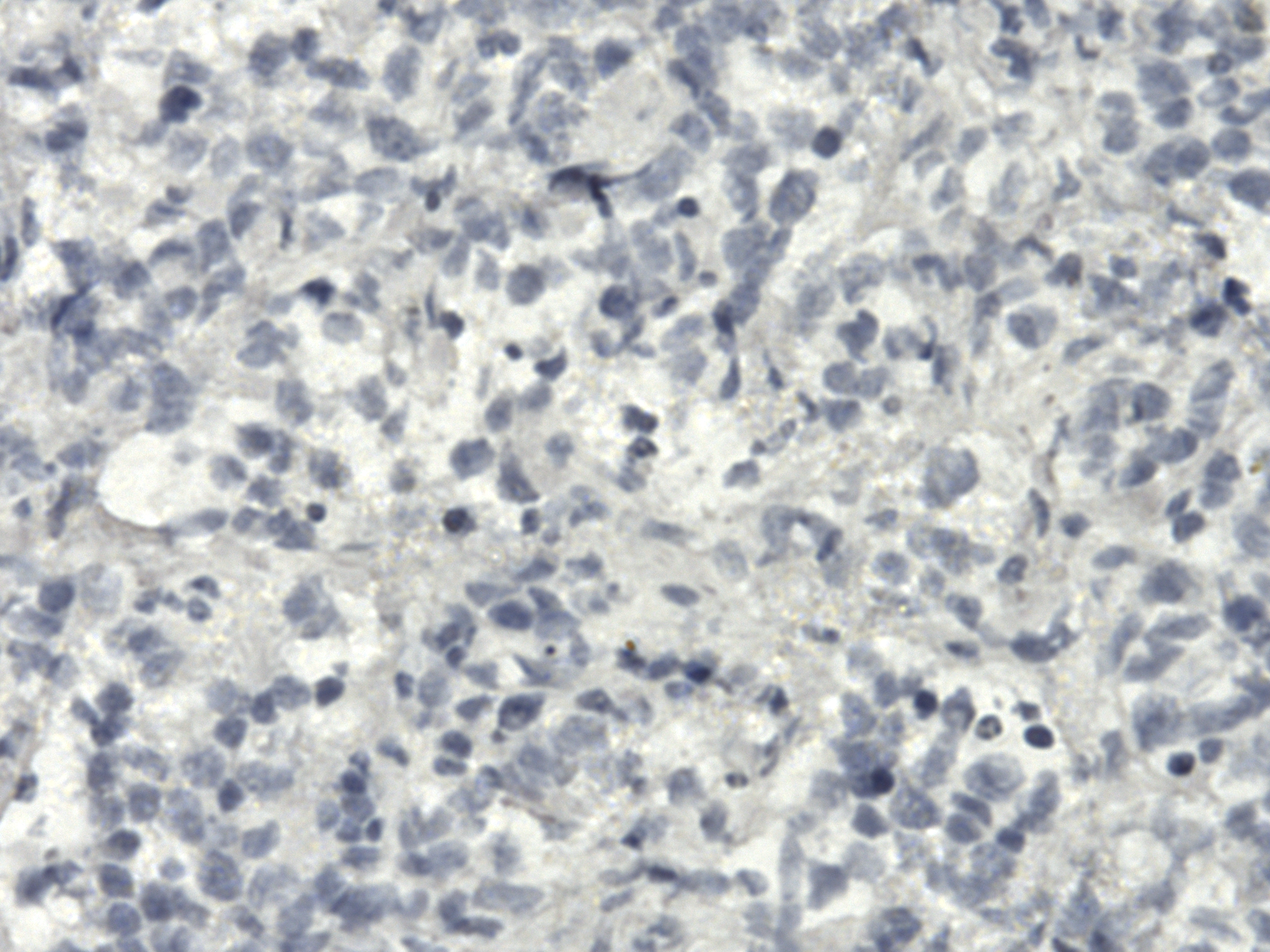

Supplement: Supplementary file 4 — Source data Fig. 2 [file 44321_2024_108_MOESM4_ESM.zip › Figure 2/2L/Ki67-DN200434.tif]

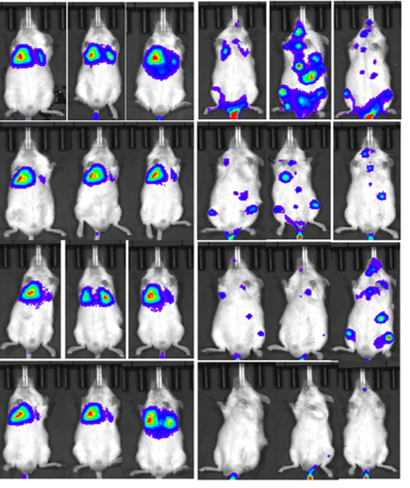

Supplement: Supplementary file 8 — Source data Fig. 6 [file 44321_2024_108_MOESM8_ESM.zip › Figure 6/6F/6F-luminescence image.tif]

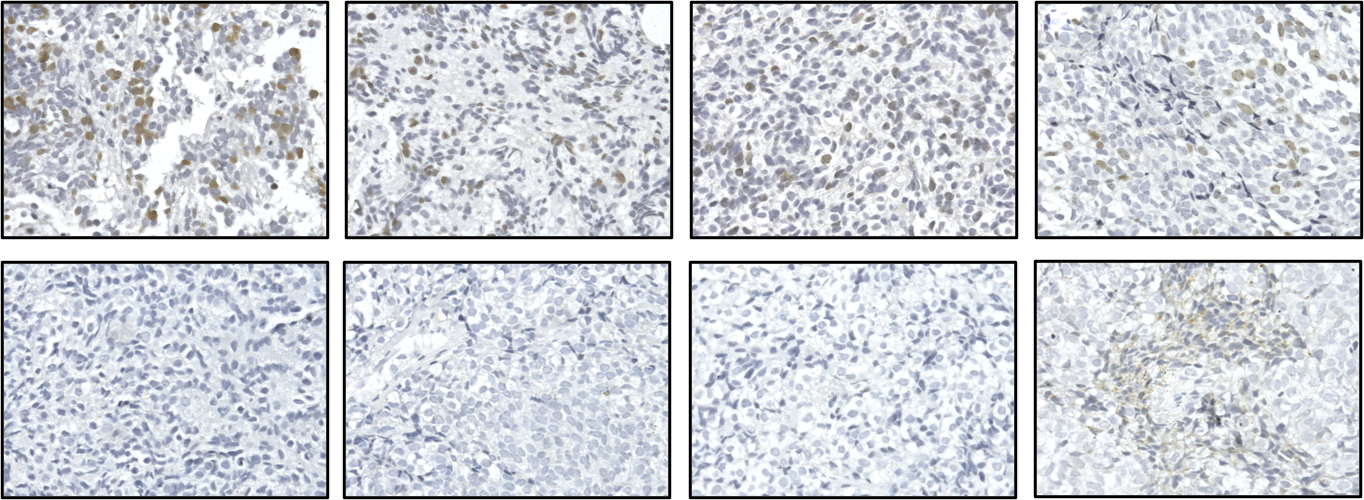

Supplement: Supplementary file 8 — Source data Fig. 6 [file 44321_2024_108_MOESM8_ESM.zip › Figure 6/6I/6I-image.tif]

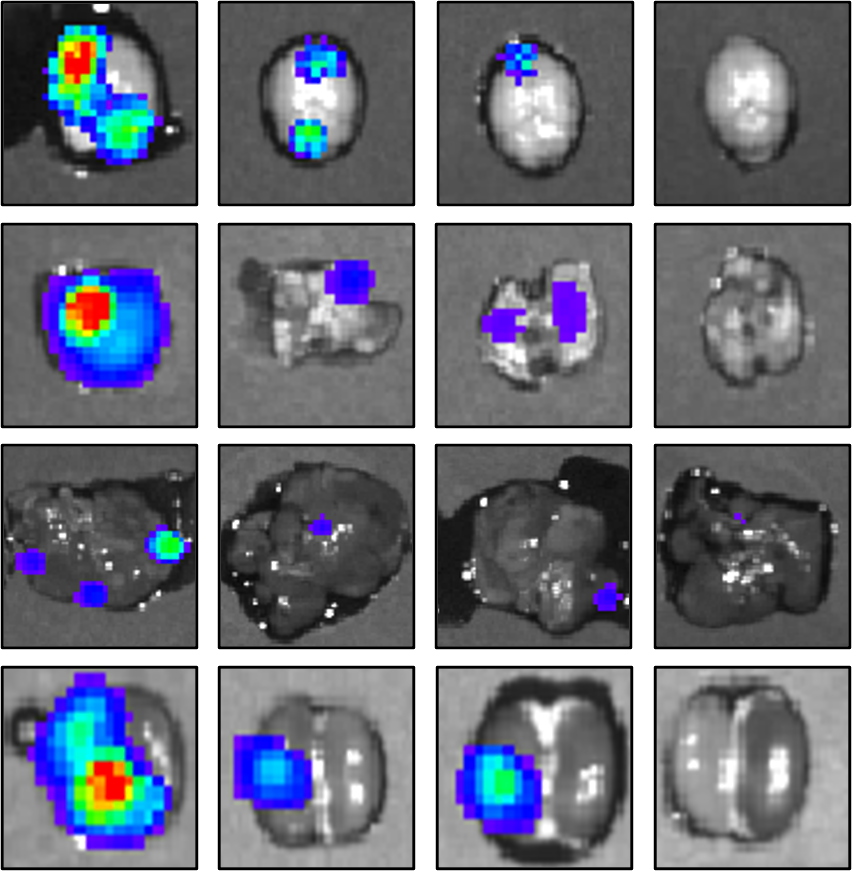

Supplement: Supplementary file 8 — Source data Fig. 6 [file 44321_2024_108_MOESM8_ESM.zip › Figure 6/6G/6G-luminescence image.tif]

Figure 6D

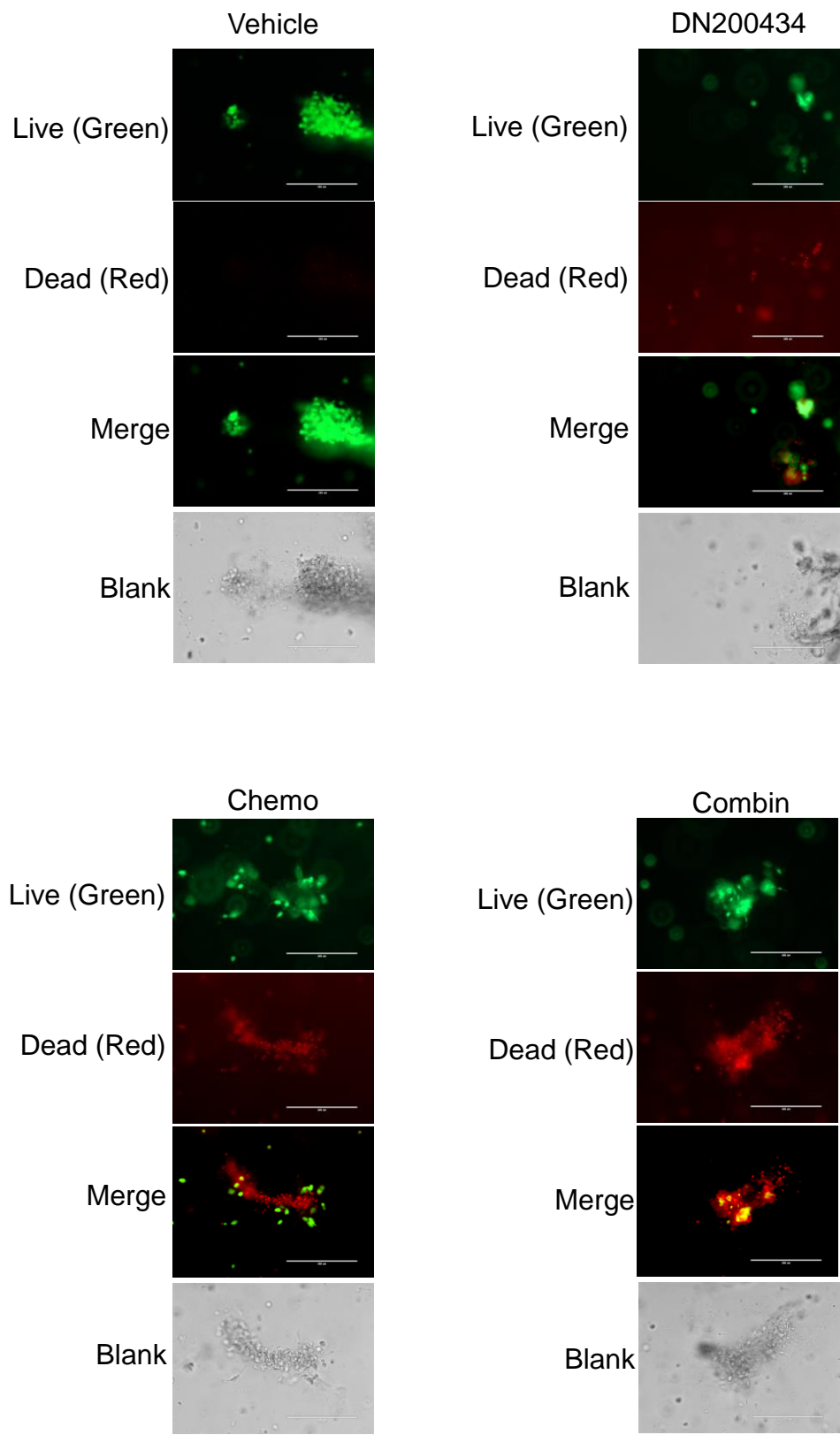

Supplement: Supplementary file 8 — Source data Fig. 6 [file 44321_2024_108_MOESM8_ESM.zip › Figure 6/6D/Figure 6D.pdf]
